# Testosterone and dihydrotestosterone reduce platelet activation and reactivity in older men and women

**DOI:** 10.18632/aging.101438

**Published:** 2018-05-02

**Authors:** Kamil Karolczak, Lucyna Konieczna, Tomasz Kostka, Piotr J. Witas, Bartlomiej Soltysik, Tomasz Baczek, Cezary Watala

**Affiliations:** 1 Department of Haemostatic Disorders, Medical University, Lodz, Poland; 2 Department of Pharmaceutical Chemistry, Medical University of Gdansk, Gdansk, Poland; 3 Department of Geriatrics, Healthy Ageing Research Centre (HARC), Medical University, Lodz, Poland

**Keywords:** blood platelets, sex steroids, atherosclerosis, aging

## Abstract

The cardiovascular effects of testosterone and dihydrotestosterone are generally attributed to their modulatory action on lipid and glucose metabolism. However, no *ex vivo* studies suggest that circulating androgen levels influence the activation and reactivity of blood platelets – one of the main components of the haemostasis system directly involved in atherosclerosis. The levels of testosterone, dihydrotestosterone and oestradiol in plasma from men and women aged from 60 to 65 years were measured by LC-MS; the aim was to identify any potential relationships between sex steroid levels and the markers of platelet activation (surface membrane expression of GPII/IIIa complex and P-selectin) and platelet reactivity in response to arachidonate, collagen or ADP, monitored with whole blood aggregometry and flow cytometry. The results of the *ex vivo* part of the study indicate that the concentrations of testosterone and its reduced form, dihydrotestosterone are significantly negatively associated with platelet activation and reactivity. These observations were confirmed in an *in vitro* model: testosterone and dihydrotestosterone significantly inhibited platelet aggregation triggered by arachidonate or collagen. Our findings indicate that testosterone and dihydrotestosterone are significant haemostatic steroids with inhibitory action on blood platelets in older people.

## Introduction

Testosterone (T) has recently been identified as a cardiovascular hormone, in addition to its basic role in the regulation of male reproduction [1,2].

The risk of cardiovascular events increases with age, and this is paralleled by the declined levels of circulating T in aging men. However, this decline is observed only in the part of the elderly male population and is not as common as widely believed [3].

In addition, some men undergoing androgen deprivation therapy due to prostate cancer present an increased risk of impaired glucose and/or lipid metabolism, often contributing to cardiovascular ischaemic events [4]. This implies that T may be somehow involved in carbohydrate and lipid metabolism and thus, may indirectly contribute to the ongoing onset of atherogenesis. Furthermore, the subpopulation of men suffering from type 2 diabetes mellitus and/or obesity, which are widely recognized as factors perpetuating atherosclerosis, also exhibit decreased levels of T, which is associated with the severity of hyperglycaemia and obesity [5].

It is believed that normal plasma concentrations of T facilitate a beneficial atherogenic lipid profile by lowering the levels of total- and LDL-cholesterol [6].

Importantly, T is a potent vasorelaxing factor, acting in both an endothelium-dependent and an endothelium-independent way. The former mechanism assumes that T enhances the expression of endothelial nitric oxide synthase, leading to the increased synthesis of nitric oxide - a strong vasorelaxing factor. The latter mechanism proposes that T affects the polarization state of smooth muscle cell membranes, thus facilitating the augmented vasorelaxing action of NO [7]. These observations imply that lower concentrations of T may be regarded as an independent factor of cardiovascular risk in men of different ages [8,9] and that the normalization of T concentrations with hormonal replacement therapy (HRT) exerts protective effects on cardiovascular system in men [10].

It is important to note that T, obviously regarded as a male cardiovascular hormone, is also present in women, where it may also have a significant beneficial impact on the cardiovascular system, together with oestrogens. Although this field has been poorly investigated [11], quite recent results indicate a significant decrease of androgen levels after menopause [12], and a higher incidence of cardiovascular events has been observed in women with lower testosteronaemia [13].

Otherwise, the role(s) of the reduced form of T, dihydrotestosterone (DHT) in the cardiovascular system, has not been widely recognised.

The question of T- and DHT-dependent sensitivity of blood platelets, the pivotal cellular players in physiological and pathological haemostasis, still remains unanswered. Only occasional *in vitro* studies have been performed in this area. They suggest that blood platelets demonstrate potential responsiveness to T, which implies that T could modulate cardiovascular risk through its influence on blood platelets. The available findings are contradictory. It has been suggested that T augments platelet reactivity through the increased expression of arachidonate receptors on blood platelets [14]. However, it has also been observed that T indirectly decreases platelet activation due to the antiplatelet action of nitric oxide (NO) secreted by T-stimulated endothelium [15].

Due to limited data on the potential interactions of T with blood platelets, it seems currently impossible to collate the *in vivo* results evaluating any associations between the markers of platelet activation and reactivity in circulating blood with the concentrations of T in peripheral blood plasma. To date, no evaluation of the relationship between the plasma levels of androgens (T and DHT) and markers of functional state of blood platelets has been performed in men or women.

Therefore the aim of the present study is to determine whether the levels of free T and DHT in circulating plasma correlate with selected markers of platelet reactivity and activation. It uses HPLC-MS to estimate the levels of steroid hormones, including T, DHT and oestradiol (E_2_). Whole blood platelet aggregability was used to evaluate the markers of platelet functional state in response to *in vitro* treatment with arachidonic acid, ADP or collagen. It also measures the expression of the active form of fibrinogen receptor (GPIIb/IIIa) and P-selectin on blood platelet surface membranes, wich are the markers of platelet readiness to aggregate and degranulate, respectively. All the above tests of platelet responsiveness to agonists (arachidonate, collagen, ADP) use typical markers of platelet reactivity. Moreover, the expression of GPIIb/IIIa and P-selectin on non-stimulated platelets reflect platelet priming in circulation and correspond to the functional state of platelets in circulation (*in vivo*).

In addition to determining whether the main male androgens are negative regulators of platelet activation and reactivity, the study also analyses relationships between plasma levels of T and DHT and selected easily-accessible biochemical and haemostatic factors in blood plasma: sub-fractions of cholesterol, triglycerides, glucose, uric acid and homocysteine (Hcy). Some of these factors are commonly recognized, while others are thought to have pro- or anti-atherogenic effects.

The study also examines whether the *in vitro* effects of T, DHT and E_2_ on the reactivity of blood platelets remain consistent with the associations revealed in the *in vivo* part of the study. All experiments were performed using the blood of males and females aged 60–65 years, in whom the cardiovascular risk may be considered higher than in younger populations. This approach also highlights the differences found in other variables associated with the levels of circulating sex steroids.

## RESULTS

### Design of the data analyses

Due to a relatively great abundance of the outcomes of the performed statistical analyses, they are presented as a hierarchy, *i.e.* starting from univariate and bivariate analyses (inference tests and simple correlations), primarily used to get a general idea of possible relationships, to multivariate analyses, which are employed to further explore the revealed links and to adjust for the presence of other covariates and confounders.

### Comparisons between men and women

Table 1 lists selected aspects of blood morphology, as well as biochemical variables, determined in serum or plasma from men and women. It shows that numerous variables differed significantly between the sexes. The use of the bootstrap–boosted approach the analysis of covariance (ANCOVA) (performed on the Box-Cox-transformed data) revealed that men demonstrated significantly higher values for variables describing blood platelets than women. Total cholesterol and its lipoprotein fractions were reduced, while uric acid and Hcy increased in the serum or plasma taken from men. All the hallmarks of blood platelet reactivity in response to agonists, measured with the use of whole blood impedance aggregometry, were elevated in women. The plasma concentrations of androgens (T and DHT) remained significantly higher in plasma samples from men (Table 1).

**Table 1:** Biochemical, platelet function, social, anthropometric and medical characteristics of investigated subjects.

**Parameter**	**Both sexes (*n* = 155)**	**Males (*n* = 73)**	**Females (*n* = 82)**
			
*Blood morphology and biochemistry*			
WBC (10^3^/mm^3^)	5.7 (5.1 – 6.9)	5.8 (5.1 – 6.9)	5.6 (5.1 – 6.8)
RBC (10^6^/mm^3^)	4.5 (4.3 – 4.7)	4.6 (4.2 – 4.9)	4.4 (4.2 – 4.6)^U††^
HGB (g/dl)	13.8 (13.0 – 14.5)	14.3 (13.7 – 14.9)	13.1 (12.6 – 13.8)^U††^
HCT (%)	39.7 (37.8 – 41.3)	40.9 (39.2 – 42.6)	38.6 (36.6 – 39.9) ^U††^
PLT (10^3^/mm^3^)	209.0 (180.0 – 241.0)	197.1 (168.6 – 228.6)	223.4 (191.9 – 242.9) ^*^
MPV (µm^3^)	11.3 (10.7 – 12.1)	11.2 (10.6 – 11.9)	11.5 (10.9 – 12.3) ^U*^
PCT (%)	0.24 (0.21 – 0.27)	0.22 (0.19 – 0.25)	0.26 (0.22 – 0.29) ^U††^
PDW (fl)	13.7 (12.4 – 15.7)	13.4 (12.1 – 15.2)	14.2 (12.7 – 16.3) ^U*^
P-LCR (%)	36.4 ± 8.1	34.9 ± 7.4	37.8 ± 8.4 ^T*^
Lym (10^3^/mm^3^)	1.89 (1.52 – 2.33)	1.81 (1.55 – 2.29)	1.93 (1.49 – 2.34)
Mono (10^3^/mm^3^)	0.52 (0.43 – 0.64)	0.56 (0.46 – 0.69)	0.49 (0.40 – 0.58) ^U#^
Neu (10^3^/mm^3^)	3.11 (2.50 – 3.79)	3.19 (2.44 – 3.77)	3.02 (2.50 – 3.83)
Eo (10^3^/mm3)	0.14 (0.10 – 0.22)	0.15 (0.11 – 0.24)	0.14 (0.09 – 0.21)
Baso (10^3^/mm^3^)	0.02 (0.02 – 0.03)	0.03 (0.02 – 0.04)	0.02 (0.02 – 0.03)
total cholesterol (mg/dl)	208.7 (174.4 – 239.5)	187.8 (169.1 – 218.8)	225.7 (182.2 – 249.4) ^U††^
triglycerides (mg/dl)	112 (78.4 – 158.7)	111.7 (77.7 – 141.9)	112.6 (78.1 – 163.7)
HDL cholesterol (mg/dl)	49 (41.5 – 60.6)	45.6 (40.2 – 51.0)	54.2 (44.1 – 63.4) ^U††^
LDL cholesterol (mg/dl)	131.5 (105.6 – 155.7)	116.3 (101.9 – 139.8)	141.2 (109.7 – 167.2) ^U†^
glucose (mg/dl)	99 (90.3 – 107.8)	100.3 (93.8 – 111.6)	96.0 (89.0 – 105.4)
uric acid (mg/dl)	4.9 (4.1 - 5.8)	5.4 (4.8 – 6.1)	4.3 (3.8 – 5.2) ^U††^
homocysteine (µmol/l)	15.1 (12.9 – 17.4)	15.7 (13.3 – 19.4)	14.3 (12.7 – 16.2) ^U#^
testosterone (ng/ml)	3.59 (2.93 – 4.49)	4.49 (4.41 – 4.71)	2.93 (2.86 – 2.99) ^U††^
dihydrotestosterone (ng/ml)	0.47 (0.32 – 0.63)	0.63 (0.62 – 0.66)	0.32 (0.32 – 0.33) ^U††^
oestradiol (ng/ml)	0.04 (0.03 – 0.05)	0.04 (0.03 – 0.05)	0.04 (0.03 – 0.05)
			
*Blood platelet activation*			
P-selectin_resting plt_ (%)	2.4 (1.5 – 4.7)	2.8 (1.7 - 4.9)	2.0 (1.4 - 4.6)
GPIIb/IIIa_resting plt_ (%)	1.9 (1.0 – 3.6)	1.7 (0.9 - 3.0)	2.2 (1.1 - 4.1) ^U*^
‘cumulative platelet activation’	0.1 (-1.4 – 1.2)	-1.6 (-4.8 - 2.7)	0.3 (-1.5 - 1.5) ^U*^
*Blood platelet reactivity* (*in vitro* response to agonists)			
A_max, arachidonate_ [a.u.]	127.9 (98.3 – 145.1)	118.8 (85.6 - 135.1)	134.4 (110.5 - 153.4) ^U††^
A_max, collagen_ [a.u.]	152.3 (121.5 – 173.6)	140.0 (114.6 - 162.8)	161.7 (132.7 - 187.3) ^U†^
A_max, ADP_ [a.u.]	124.1 (103.1 – 143.3)	111.3 (92.6 - 128.6)	133.1 (120.1 - 154.0) ^U†††^
‘cumulative blood platelet reactivity’	0.1 (-1.9 – 1.9)	-1.0 (-2.3 - 0.1)	1.2 (-1.3 - 2.8) ^U†††^
P-selectin_arachidonate_ [%]	31.3 (13.3 – 49.9)	26.2 (12.8 – 40.3)	42.8 (31.0 - 55.6)^U*^
P-selectin_collagen_ [%]	25.6 (11.9 – 47.6)	23.4 (12.2 – 41.4)	26.2 (11.7 – 48.8)
GPIIb/IIIa_arachidonate_ [%]	30.0 (16.1 – 51.6)	25.1 (12.8 – 45.5)	37.3 (17.1 – 55.4) ^U*^
GPIIb/IIIa_collagen_ [%]	37.2 (15.0 – 53.8)	34.9 (13.4 – 52.3)	39.6 (15.7 – 57.4)
‘cumulative P-selectin expression’	-0.3 (-1.2 – 1.0)	-0.1 (-0.1 - 0.1)	0.1 (-1.1 - 1.1)
‘cumulative GPIIb/IIIa expression’	-0.1 (-1.5 – 1.0)	-0.5 (-2.3 - 1.1)	0.3 (-1.4 - 1.3)
			
*Social and anthropometric indicators*			
age [years]	62. 7 ± 1.7	62.9 ± 1.7	62.4 ± 3.6
BMI [kg/m^2^]	28.2 ±4.7	27.9 ± 4.2	28.4 ± 5.1
WHR	0.92 ± 0.10	0.99 ± 0.05	0.86 ± 0.09
current smoking	19	23	16
			
*Medical indicators*			
hypertension [%]	49	55	44
hypercholesterolaemia [%]	64	49	76
type 2 diabetes mellitus [%]	10	8	11
myocardial infarction in the past [%]	3	4	1
stroke in the past [%]	3	3	2
cancer in the past [%]	4	3	6
depression [%]	14	8	19
			
*Current therapy*			
*- cardiovascular drugs* [%] (incl. beta-blockers and nitrates)	25.3	21.9	28.9
*- antihypertensive drugs* [%] (incl. ACE inhibitors, sartans, Ca^2+^ channel blockers, alpha-blockers)	32.5	35.6	30.1
*- antihyperlipidemic drugs* [%] (incl. statins and fibrates)	24.7	30.1	20.5
*- antidiabetic drugs* [%] (incl. insulin, metformin and gliclazide)	10.4	9.6	10.8
*- other drugs* [%] (incl. SAID, NSAID, H_2_ receptor blockers, antihistamines, antidepressants, beta-mimetics)	31.8	19.2	43.4

Variables are presented as means ± SD, medians with interquartile ranges or percentage fractions of whole groups of investigated patients. All continuous variables but age were adjusted for age in separate groups of men and women.

Comparisons between the groups were performed for adjusted values with the use of covariance analysis (ANCOVA) on the Box-Cox-transformed data: ^*^P≤ 0.05; ^#^*P*< 0.01; ^†^*P*< 0.005; ^††^*P*< 0.001; ^†††^*P*<< 0.0001.

Abbreviations used: ACE, angiotensinogen converting enzyme; ADP, adenosine diphosphate; A_max_, maximal aggregation of blood platelets; ASA, acetylsalicylic acid; Baso, number of basophils; BMI, body mass index; Eo, number of eosinophils; GPIIb/IIIa, the glycoprotein complex in platelet surface membranes, the receptor for fibrinogen; HCT, haematocrit; H_2_ receptor, histamine receptor H_2_; HDL, high density lipoproteins; HGB, concentration of haemoglobin; LDL, low density lipoproteins; LYM, number of lymphocytes; Mono, number of monocytes; MPV, mean platelet volume; Neu, number of neutrophils; NSAID, non-steroid anti-inflammatory drugs; PCT, plateletcrit; PDW, platelet distribution width; P-LCR, platelet-large cells ratio; PLT/plt, platelet count/platelets;; RBC, red blood cell count; SAID, steroid anti-inflammatory drugs; WBC, white blood cell count; WHR, waist-hip ratio.

### Comparisons between individuals with lower and higher blood platelet reactivity

The dichotomised values of the van der Waerden normal scores of A_max_ cumulated through the agonists used (AA, collagen, ADP), referred to as ‘cumulative blood platelet reactivity’, were used to discriminate individuals with lower or higher blood platelet reactivity. The characteristics of blood morphology and biochemistry, and those of the functional state of blood platelets in subjects with lower or higher blood platelet reactivity, are given in Tables 2 and 3, respectively. The individuals with higher ‘cumulative blood platelet reactivity’ were characterized by very significantly elevated PLT and PCT, as well as increased MPV and P-LCR. Blood haematocrit was slightly, but significantly reduced, leukocyte count and neutrophil count remained higher (*P*< 0.05), whereas serum concentration of uric acid, T and DHT were reduced in this group. As expected, the blood platelet response to agonists was significantly increased in the group with higher ‘cumulative blood platelet reactivity’. Otherwise, the elevations in two surface membrane markers, P-selectin and the activated GPIIb/IIIa, appeared non-significant, although their cumulative measures (‘cumulative platelet activation’, as well as the agonist-induced ‘cumulative P-selectin expression’ and ‘cumulative GPIIb/IIIa expression’) demonstrated significantly increased values in the individuals with higher platelet reactivity (Tables 2 and 3).

**Table 2:** Morphological and biochemical characteristics of subjects with lower and higher ‘cumulative blood platelet reactivity’.

**Parameter**	**Individuals with lower ‘cumulative blood platelet reactivity’** **(*n*= 77)**	**Individuals with higher ‘cumulative blood platelet reactivity’** **(*n*= 78)**
age (years)	63 (62 - 65)	62 (61 - 64)
men [%]	64.9	29.5
WBC (10^3^/mm^3^)	5.6 (4.8 – 6.4)	5.8 (5.2 – 7.0) ^U*^
RBC (10^3^/mm^3^)	4.5 ± 0.4	4.5 ± 0.4
HGB (g/dl)	14.0 ± 1.1	13.8 ± 1.0
HCT (%)	40.1 ± 2.9	39.3 ± 2.6 ^U*^
PLT (10^3^/mm^3^)	196.1 ± 36.9	228.7 ± 47.8 ^T†††^
MPV (µm^3^)	11.1 ± 0.9	11.5 ± 0.1 ^T*^
PCT (%)	0.22 ± 0.04	0.26 ± 0.05 ^T†††^
P-LCR (%)	33.9 ± 7.6	37.2 ± 7.8 ^T*^
PDW	12.9 (12.0 – 15.3)	13.8 (12.6 – 15.9)
Lym (10^3^/mm^3^)	1.72 (1.46 – 2.23)	2.02 (1.57 – 2.43)
Mono (10^3^/mm^3^)	0.52 (0.44 – 0.62)	0.53 (0.42 – 0.65)
Neu (10^3^/mm^3^)	3.10 (2.47 – 3.68)	3.14 (2.61 – 3.95) ^U*^
Eo (10^3^/mm^3^)	0.14 (0.10 – 0.24)	0.15 (0.10 – 0.22)
Baso (10^3^/mm^3^)	0.03 (0.02 – 0.03)	0.02 (0.02 – 0.03)
total cholesterol (mg/dl)	206.0 (171.8 – 244.2)	211.5 (178.9 – 241.7)
triglycerides (mg/dl)	113.9 (77.6 – 162.0)	108.6 (82.5 – 161.9)
HDL cholesterol (mg/dl)	47.3 (41.4 – 54.0)	52.3 (42.6 – 63.6)
LDL cholesterol (mg/dl)	130.3 ± 38.8	136.8 ± 35.1
glucose (mg/dl)	99.2 (90.1 – 107.8)	97.2 (90.3 – 105.6)
uric acid (mg/dl)	5.3 ± 1.3	4.8 ± 1.1 ^T††^
homocysteine (µmol/l)	14.3 (12.4 – 19.2)	15.2 (13.2 – 17.0)
testosterone (ng/ml)	4.5 (3.1 – 4.6)	2.9 (2.9 – 4.4) ^U†††^
dihydrotestosterone (ng/ml)	0.6 (0.3 – 0.7)	0.3 (0.3 – 0.6) ^U†††^
oestradiol (ng/ml)	0.04 (0.03 – 0.06)	0.04 (0.03 - 0.05)

Variables, adjusted for age and sex, presented as means ± SD, medians with interquartile ranges or percent fractions of whole groups of investigated patients. The individuals with lower or higher ‘cumulative blood platelet reactivity’ were identified according to the dichotomised values of the van der Waerden normal scores of A_max_ cumulated through the agonists used (AA, collagen, ADP), referred to as ‘cumulative blood platelet reactivity’ (see details in *Materials and methods* and *Results*).

Comparisons between the groups were performed on the Box-Cox-transformed adjusted values with the use of covariance analysis (ANCOVA): ^*^P≤ 0.05; ^#^*P*< 0.01; ^†^*P*< 0.005; ^††^*P*< 0.001; ^†††^*P*<< 0.0001.

Abbreviations used: Baso, number of basophils; BMI, body mass index; Eo, number of eosinophils; HCT, haematocrit; HDL, high density lipoproteins; HGB, concentration of haemoglobin; LDL, low density lipoproteins; LYM, number of lymphocytes; Mono, number of monocytes; MPV, mean platelet volume; Neu, number of neutrophils; PCT, plateletcrit; PDW, platelet distribution width; P-LCR, platelet-large cells ratio; PLT, platelet count; RBC, red blood cell count; WBC, white blood cell count.

**Table 3:** Blood platelet activation and reactivity characteristics of subjects with lower and higher ‘cumulative reactivity’ of blood platelets.

**Parameter**	**Individuals with lower ‘cumulative blood platelet reactivity’** **(*n*= 77)**	**Individuals with higher ‘cumulative blood platelet reactivity’** **(*n*= 78)**
*platelet activation* (in circulating/resting platelets)		
P-selectin_resting plt_ (%)	2.5 (1.4 – 4.6)	2.3 (1.6 – 4.7)
GPIIb/IIIa _resting plt_ (%)	1.7 (0.8 – 3.1)	2.2 (1.1 – 4.0)
‘cumulative platelet activation’	-1.0 (-5.2 – 0.7)	0.3 (-1.6 – 1.7)^U*^
*platelet reactivity* (*in vitro* response to agonists)		
A_max, arachidonate_ [a.u.]	104.1 (78.0 – 121.5)	142.4 (131.0 – 157.6) ^U†††^
A_max, collagen_ [a.u.]	123.6 (93.9 – 140.0)	173.3 (157.9 – 191.0) ^U†††^
A_max, ADP_ [a.u.]	97.1 ± 22.9	139.5 ± 20.6 ^T†††^
‘cumulative blood platelet reactivity’	-1.9 (-2.8 - -1.0)	1.8 (1.0 – 3.5) ^U†††^
P-selectin_arachidonate_ [%]	44.3 (31.3 – 62.5)	46.9 (31.2 – 62.4) ^U#^
P-selectin_collagen_ [%]	45.8 (28.1 – 66.5)	47.7 (30.1 – 59.5)
GPIIb/IIIa _arachidonate_ [%]	43.5 (26.2 – 60.0)	46.3 (35.3 – 61.2) ^U#^
GPIIb/IIIa_collagen_ [%]	51.2 (40.3 – 57.7)	49.6 (36.8 – 68.4) ^U#^
‘cumulative P-selectin expression’	-0.4 (-1.2 – 0.1)	0.2 (-0.9 – 1.2) ^U#^
‘cumulative GPIIb/IIIa expression’	-0.8 (-2.4 – 0.5)	0.4 (-1.2 – 1.3) ^U#^
		

Variables, adjusted for age and sex, are presented as means ± SD or medians with interquartile ranges. The individuals with ‘lower’ or ‘higher blood platelet reactivity’, were identified according to the dichotomised values of the van der Waerden normal scores of A_max_ cumulated through the agonists used (AA, collagen, ADP), referred to as ‘cumulative blood platelet reactivity’. The values of the van der Waerden normal scores of the expressions of surface membrane antigens in circulating resting platelets cumulated through the antigens (P-selectin or the active GPIIb/IIIa) are referred to as ‘cumulative platelet activation’ and the van der Waerden normal scores of platelet response to agonists cumulated through the agonists used (AA, collagen) are referred to as ‘cumulative P-selectin/GPIIb/IIIa expression’ (see details in *Materials and methods* and *Results*).

Comparisons between the groups were performed on the Box-Cox-transformed adjusted values with the use of the covariance analysis (ANCOVA): ^*^P≤ 0.05; ^#^*P*< 0.01; ^†^*P*< 0.005; ^††^*P*< 0.001; ^†††^*P*<< 0.0001.

Abbreviations used: ADP, adenosine diphosphate; A_max_, maximal aggregation of blood platelets; GPIIb/IIIa, the glycoprotein complex in platelet surface membranes, the receptor for fibrinogen.

### Bivariate association analyses: androgens and blood morphology

The nonparametric correlation analysis was performed among the whole group of patients, including both sexes. A simple bivariate association analysis was performed, either not taking into account the possible confounders and accompanying variables, or with an adjustment for sex (partial Spearman’s correlation). While simple association analysis without adjustment demonstrated that plasma testosteronaemia was very significantly and positively associated with the variables of red blood cell morphology, including red blood cell count (*R*_S LOO_= 0.336; *P*<< 0.0001), haemoglobin concentration (*R*_S LOO_ = 0.524; *P*<< 0.0001) and blood haematocrit (*R*_S LOO_ = 0.460; *P*<< 0.0001), the adjustment for sex revealed that partial Spearman’s correlation coefficients became considerably reduced, and were much below the level of statistical significance: RBC (*R*_S LOO_= 0.020; *NS*), HGB (*R*_S LOO_ = 0.055; *NS*) and HCT (*R*_S LOO_ = 0.095; *NS*). Similarly, in a simple correlation analysis without adjustment, plasma levels of DHT were also significantly and positively associated with the variables related to erythrocyte morphology, including red blood cell count (*R*_S LOO_ = 0.320; *P*<< 0.0001), haemoglobin concentration (*R*_S LOO_ = 0.494; *P*<< 0.0001) and haematocrit (*R*_S LOO_ = 0.426; *P*<< 0.0001), and again, these significant associations disappeared upon adjusting for sex (*R*_S LOO_RBC_ = 0.005, *R*_S LOO_HGB_ = 0.016 and *R*_S LOO_HCT_ = 0.044, respectively; all *NS*).

On the contrary, plasma concentrations of T and DHT appeared very significantly negatively associated with the morphology of blood platelets for non-adjusted analysis. These significant relationships disappeared for all variables, but not for MPV and P-LCR, upon adjusting for sex (PLT: RS,LOO_T PLT= -0.215, PT PLT< 0.01 and RS,LOO_T PLT_sex-adjusted= -0.011, NS; RS,LOO_DHT PLT= -0.217, PDHT PLT< 0.01 and RS,LOO_DHT PLT_sex-adjusted= -0.007, NS; MPV: (RS,LOO_T MPV= -0.212, PT MPV< 0.01 and RS LOO_T MPV_sex-adjusted= -0.119, PT PLT= 0.070; RS,LOO_DHT MPV= -0.227, PDHT MPV< 0.005 and RS,LOO_DHT MPV_sex-adjusted= -0.147; PDHT MPV= 0.033; PCT: RS,LOO_T PCT= -0.305, PT PCT< 0.0001 and RS,LOO_T PCT_sex-adjusted= -0.002, NS; RS,LOO_DHT PCT= -0.311, PDHT PCT< 0.0001 and RS,LOO_DHT PCT_sex-adjusted= -0.028; NS; PDW: RS,LOO_T PDW= -0.189, PT< 0.02 and RS,LOO_T PDW_sex-adjusted= -0.085; NS; RS,LOO_DHT PDW= -0.199; PDHT PDW< 0.02 and RS,LOO_DHT PDW_sex-adjusted= -0.104, NS; P-LCR: RS,LOO_T P-LCR= -0.230; PT P-LCR< 0.005 and RS,LOO_DHT P-LCR_sex-adjusted= -0.141; PT P-LCR= 0.040; RS,LOO_DHT P-LCR= -0.246; PDHT P-LCR< 0.002 and RS,LOO_DHT_sex-adjusted= -0.170; PDHT P-LCR< 0.02). 

No significant associations were found between the plasma concentrations of androgens and white blood cell morphology, or between the parameters of platelet or erythrocyte morphology and plasma concentration of E_2_ (data not shown).

In addition, the variables describing blood platelet morphology demonstrated significant positive associations with selected markers of platelet reactivity. The most significantly associated were: platelet count (*R*_S LOO_Amax_arachidonate_=0.284/*R*_S LOO_Amax_arachidonate,sex-adjusted_=0.231, *P*=0.0002/0.002; *R*_S LOO_Amax_collagen_=0.279/*R*_S LOO_Amax_collagen,sex-adjusted_=0.231, *P*=0.0002/0.002; *R*_S LOO_Amax_ADP_=0.402/*R*_S LOO_Amax_ADP,sex-adjusted_=0.331, *P*<<0.0001/<<0.0001; *R*_S LOO_cumulative plt reactivity_=0.354/*R*_S LOO_cumulative plt reactivity,sex-adjusted_=0.294, *P*<<0.0001/0.0001), MPV (*R*_S LOO_Amax_arachidonate_=0.142, *P*=0.036; *R*_S LOO_Amax_ADP_ =0.216/*R*_S LOO_Amax_ADP,sex-adjusted_=0.216/0.159, *P*=0.003/0.024; *R*_S LOO_cumulative plt reactivity_=0.178/ *R*_S LOO_cumulative plt reactivity,sex-adjusted_=0.178/0.123, *P*=0.013/0.061; *R*_S LOO_GPIIbIIIa_arachidonate_=0.190, *P*=0.011; *R*_S LOO_GPIIbIIIa_collagen_=0.176, *P*=0.016) and the marker of large circulating platelets, P-LCR (*R*_S LOO_Amax_arachidonate_=0.154, *P*=0.029; *R*_S LOO_Amax_ADP_=0.233/*R*_S LOO_Amax_ADP,sex-adjusted_=0.171, *P*=0.002/0.016; *R*_S LOO_cumulative plt reactivity_=0.192/*R*_S LOO_cumulative plt reactivity,sex-adjusted_=0.136, *P*=0.008/0.046; *R*_S LOO_GPIIbIIIa_arachidonate_=0.201, *P*=0.007; *R*_S LOO_GPIIbIIIa_collagen_ =0.178, *P* = 0.016).

### Associations of testosterone and dihydrotestosterone levels with selected plasma markers of atherogenesis – bivariate analyses

For the whole group, non-adjusted for confounding variables, plasma levels of T and DHT were significantly associated with blood plasma concentrations of several solutes, some of which being commonly acknowledged as pro- or anti-atherogenic factors. Negative associations were found between T or DHT and the concentrations of cholesterol subfractions, but only when the estimation was performed for both sexes analysed together (Table 4).

**Table 4:** Associations between selected markers of atherosclerosis and the concentrations of testosterone (T) or dihydrotestosterone (DHT) in blood serum or plasma.

**Associated variables**	**Coefficient** **of association** **(*R*_S_)**	**Value of *a posteriori* probablility** **(*P_1α_*)**
***testosterone*** total cholesterol	-0.264 (-0.056)	0.0005 (0.244)
	^m^ -0.073	0.182
	^f^ 0.115	0.077
triglycerides	-0.038 (-0.023)	0.316 (0.388)
	^m^ -0.031	0.350
	^f^ 0.054	0.251
HDL cholesterol	-0.322 (-0.035)	<<0.0001 (0.331)
	^m^ -0.073	0.182
	^f^ 0.018	0.414
LDL cholesterol	-0.177 (-0.092)	0.014 (0.127)
	^m^ -0.040	0.313
	^f^ 0.165	0.020
fasting glycaemia	0.223 (0.189)	0.003 (0.009)
	^m^ 0.241	0.001
	^f^ 0.142	0.039
uric acid	0.411 (0.220)	<<0.0001 (0.003)
	^m^ 0.141	0.040
	^f^ 0.307	<<0.0001
homocysteine	0.234 (0.040)	0.002 (0.311)
	^m^ 0.224	0.003
	^f^ -0.127	0.057
***dihydrotestosterone*** total cholesterol	-0.252 (-0.059)	0.001 (0.233)
	^m^ -0.037	0.323
	^f^ 0.096	0.118
triglycerides	-0.040 (-0.017)	0.311 (0.417)
	^m^ -0.001	0.496
	^f^ 0.042	0.304
HDL cholesterol	-0.304 (-0.016)	0.0001 (0.422)
	^m^ -0.050	0.267
	^f^ 0.025	0.378
LDL cholesterol	-0.173 (-0.083)	0.016 (0.152)
	^m^ -0.015	0.425
	^f^ 0.122	0.066
fasting glycaemia	0.225 (0.185)	0.002 (0.011)
	^m^ 0.224	0.003
	^f^ 0.158	0.025
uric acid	0.403 (0.206)	<<0.0001 (0.005)
	^m^ 0.127	0.058
	^f^ 0.333	<<0.0001
homocysteine	0.196 (0.024)	0.007 (0.383)
	^m^ 0.192	0.008
	^f^ -0.210	0.004

Associations, estimated in the group composed of 73 men and 82 women, are presented as the LOO-boosted non-adjusted or sex-adjusted Spearman’s rank correlation coefficients; the values adjusted for sex, given in parentheses, were calculated with the partial Spearman’s correlation analysis. Spearman correlation coefficients, calculated separately for men (^m^) and women (^f^), were estimated by bootstrap resampling with replacement adjusted for the sample size of the overall group (n=155) (10000 iterations). LOO, the leave-one-out (‘jackknife’ or ‘*d*-jackknife’) algorithm.

The plasma concentrations of both androgens, T and DHT, when both sexes were analysed together, were significantly positively associated with fasting glycaemia and homocysteinaemia, and a particularly significant positive correlation was revealed between uric acid and both androgens. Upon adjusting for sex, the significant relationships were maintained between both T and DHT level and fasting glycaemia and uric acid levels (Table 4).

In the excluded subgroup of women, a significant association between T and the concentration of uric acid was observed (*R*_S LOO_= 0.304; *P*< 0.005 for women and *R*_S LOO_= 0.411; *P*<< 0.0001 for the whole group), while DHT was significantly associated with the plasma concentrations of uric acid (*R*_S LOO_= 0.330; *P*< 0.003 for women and *R*_S LOO_= 0.403; *P*<< 0.0001 for the whole group) and Hcy (*R*_S LOO_= 0.196; *P*< 0.02 for the whole group). These associations were enhanced by the use of the bootstrap procedure with resampling adjusted for the sample size of the overall group (n=155),, indicating significant correlations of T with uric acid, glucose and LDL-cholesterol and of DHT with uric acid and glucose (Table 4, the data with the superscript ^f^).

For men, significant associations were found between plasma concentrations of T and fasting glycaemia (*R*_S LOO_ = 0.240; *P*< 0.05 and *R*_S LOO_= 0.223; *P*< 0.003 for the whole group) and Hcy (*R*_S LOO_= 0.234; *P*< 0.002 and *R*_S LOO_= 0.234; *P*< 0.03 for the whole group), and also between the plasma concentration of DHT and fasting glycaemia (*R*_S LOO_= 0.229; *P*< 0.05 and *R*_S LOO_= 0.225; *P*< 0.002 for the whole group). Again, when a bootstrap procedure with resampling adjusted for the sample size of the overall group (n=155) was added, significant correlations were found between T and glucose or Hcy and also between DHT and glucose or Hcy (Table 4, the data with the superscript ^m^).

E_2_ was not significantly associated with any of the above mentioned plasma markers of atherosclerosis, either in a whole group of the examined individuals or upon adjustment for sex (not shown).

### Associations of testosterone and dihydrotestosterone with the markers of platelet activation and reactivity

In the group of male and female subjects (analysed together) plasma concentrations of T and DHT remained significantly negatively associated with platelet aggregation in response to arachidonate, collagen or ADP (Table 5). Otherwise, plasma concentrations of E_2_ demonstrated no significant association with the reactivity of blood platelets agonized with arachidonate, collagen or ADP (not shown).

**Table 5:** Associations between the concentrations of testosterone (T) or dihydrotestosterone (DHT) and agonist-dependent whole blood platelet aggregation (WBA) or the expression of the markers of resting platelet activation (FC).

**Associated variable**	**Coefficient** **of association** **(*R*_S_)**	**Value of *a posteriori* probablility** **(*P_1α_*)**
***testosterone*** membrane expression of P-selectin on resting platelets	-0.146 (-0.138)	0.035 (0.043)
	^m^ -0.387	<<0.0001
	^f^ -0.144	0.037
membrane expression of GPII/IIIa on resting platelets	-0.170 (-0.120)	0.017 (0.068)
	^m^ -0.313	<<0.0001
	^f^ -0.152	0.029
AA-dependent aggregation	-0.371 (-0.287)	<<0.0001 (0.0001)
	^m^ -0.131	0.054
	^f^ -0.343	<<0.0001
COL-dependent aggregation	-0.269 (-0.173)	0.0004 (0.016)
	^m^ 0.050	0.268
	^f^ -0.185	0.011
ADP-dependent aggregation	-0.437 (-0.157)	<<0.0001 (0.026)
	^m^ -0.137	0.045
	^f^ -0.104	0.098
***dihydrotestosterone*** membrane expression of P- selectin on resting platelets	-0.162 (-0.149)	0.022 (0.032)
	^m^ -0.394	<<0.0001
	^f^ -0.150	0.031
membrane expression of GPII/IIIa on resting platelets	-0.191 (-0.154)	0.009 (0.028)
	^m^ -0.353	<<0.0001
	^f^ -0.159	0.024
AA-dependent aggregation	-0.396 (-0.326)	<<0.0001 (<<0.0001)
	^m^ -0.161	0.023
	^f^ -0.396	<<0.0001
COL-dependent aggregation	-0.292 (-0.161)	0.0001 (0.023)
	^m^ -0.104	0.099
	^f^ -0.214	0.004
ADP-dependent aggregation	-0.456 (-0.179)	<<0.0001 (0.013)
	^m^ -0.147	0.034
	^f^ -0.148	0.033

Associations, estimated in the group composed of 73 men and 82 women, are presented as the LOO-boosted non-adjusted or sex-adjusted Spearman’s rank correlation coefficients; the values adjusted for sex, given in parentheses, were calculated with the partial Spearman’s correlation analysis. Spearman’s correlation coefficients, calculated separately for men (^m^) and women (^f^), were estimated by bootstrap resampling with replacement adjusted for the sample size of the complete group (n=155) (10000 iterations). Platelet aggregation was triggered by the addition of 0.5 mM AA, 1 µg/ml collagen (COL) or 10 µM ADP and monitored with the use of a whole blood impedance aggregometry. In flow cytometry, whole blood samples stained with the gating anti-CD61 MoAb and either anti-CD62 or PAC-1 MoAb, were fixed and monitored with flow cytometry (10000 events) (for details see ‘*Materials and methods*’).

Abbreviations used: AA, arachidonic acid; ADP, adenosine diphosphate; anti-CD61 MoAb, monoclonal antibody against surface membrane GPIIIa; anti-CD62 MoAb, monoclonal antibody against surface membrane P-selectin; COL, equine tendon collagen; FC, flow cytometry; LOO, the leave-one-out (‘jackknife’ or ‘*d*-jackknife’) algorithm of the calculus; PAC-1 MoAb, monoclonal antibody against surface membrane activated complex GPIIb/GPIIIa; WBA, whole blood impedance aggregometry.

The expression of the active form of GPII/IIIa glycoprotein (receptor of fibrinogen) on the surface membranes of non-activated (resting) circulating blood platelets and *in vitro* stimulated platelets were significantly negatively associated with plasma concentrations of T or DHT (Table 5), but not E_2_ (not shown). Upon adjustment for sex, significant associations were still revealed between T or DHT level and blood platelet reactivity in response to AA, COL or ADP. Otherwise, for the extent of the activation of circulating platelets, the correlations were insignificant (T) or at the border of significance (DHT) (Table 5). The associations in separate subgroups of male and female subjects were further investigated using a resampling procedure with replacement adjusted for the overall sample size (n=155). In the men, the most significant associations were revealed between T and the activation of circulating platelets (P-selectin and the activated α_IIb_β_3_ expression) or ADP-dependent aggregation, while circulating platelet activation (both surface antigens) and platelet aggregation dependent on AA and ADP correlated most strongly with DHT. In turn, in the women, associations were found between platelet activation (both antigens) and aggregation dependent on AA and COL (for T), or between platelet activation (both antigens) and aggregation triggered by AA, COL or ADP (for DHT) (Table5, the data with the superscripts ^m^ or ^f^).

### The association between testosterone and dihydrotestosterone and the markers of platelet activation and reactivity, adjusted for plasma markers of atherogenesis and blood morphology variables – multivariate analyses

Three different multivariate approaches were employed to better characterize the associations between plasma androgen concentrations and platelet function upon adjustment for confounders: multivariate regression, logistic regression and linear discriminant analysis.

A multivariate regression analysis was used to determine the impact of confounding/co-explanatory variables on the modulation of the association(s) between the variables describing blood platelet functioning and the plasma concentrations of androgens. The following were used as dependent variables: the van der Waerden normal scores of A_max_ cumulated through the agonists used (AA, collagen, ADP), referred to as ‘cumulative blood platelet reactivity’, the van der Waerden normal scores of the surface membrane antigens (P-selectin and the active form of GPIIb/IIIa) in circulating platelets, referred to as ‘cumulative platelet activation’, or the van der Waerden normal scores of the surface platelet membrane expressions of P-selectin/the active form of GPIIb/IIIa, cumulated through the used agonists (AA, collagen), referred to as ‘cumulative P-selectin/cumulative GPIIb/IIIa expression’. The set of independent variables included age and gender, T or DHT, platelet and leukocyte counts, haemoglobin, total cholesterol, glucose, uric acid and Hcy.

The multivariate regression parameters for testosterone and the co-explanatory variables for the model of ‘cumulative blood platelet reactivity’ as a dependent variable are given in Table 6. The variables significantly contributing to the ‘cumulative blood platelet reactivity’ in the model with T were platelet count, uric acid, glucose, homocysteine and testosterone; the T level contributed mostly to explaining of the variability of the dependent variable (‘cumulative blood platelet reactivity’), as reasoned from the highest absolute value of the standardized beta coefficients. However, due to the considerable variability of T, the statistical significances of the partial correlation coefficient and semipartial correlation coefficient were rather low (*P*< 0.02 for each), which implies that other variables in the model have large compounding contributions to the association between T and ‘cumulative blood platelet reactivity’ (the univariate correlation coefficient between T and ‘cumulative blood platelet reactivity’ was *r*_P_= -0.371, *P*<< 0.0001). When the multiple regression analysis was performed separately for men and for women, the relationships revealed for the overall group were largely maintained. In men, the absolute values of the standardized beta coefficients indicated that uric acid, glucose, platelet count and Hcy contributed to the highest extent to the variability of the ‘cumulative blood platelet reactivity’ in the model with T. Amongst these contributors, uric acid and glucose were characterized by the highest significance of partial and semipartial correlation coefficients, indicating that other variables in the model contributed much less to the association between ‘cumulative blood platelet reactivity’ and uric acid or glucose. In the same model for women, platelet count, T and uric acid level contributed to the highest extent, while platelet count, uric acid, Hcy and T demonstrated the highest partial and semipartial correlation with ‘cumulative blood platelet reactivity’ (Table 6, the data with the superscripts ^m^ and ^f^). When the ‘cumulative platelet activation’ was used as a dependent variable, only haemoglobin appeared significant (resp., coeff. β: 0.251 + 0.107, *P*< 0.02), while both leukocyte count and T remained beyond statistical significance (coeff. β: -0.177 + 0.094, *P*= 0.061 and coeff. β: -0.716 + 0.403, *P*= 0.78). In a separate subgroup of men, the significant contributor was glucose (coeff. β: -0.311 + 0.085, *P*= 0.0004), and in a subgroup of women, it was Hcy (coeff. β: -0.288 + 0.083, *P*= 0.0007). For the van der Waerden normal scores of the ‘cumulative GPIIb/IIIa expression’ as a dependent variable, only Hcy remained statistically significant (coeff. β: -0.180 + 0.090, *P*< 0.05), while T was beyond significance (resp. coeff. β: -0.680 + 0.403, *P*= 0.094) in the overall group of patients. In men, Hcy and glucose (coeff. β_Hcy_: -0.330 + 0.088, *P*< 0.0002 and coeff. β_glucose_: -0.244 + 0.087, *P*< 0.005) were significant contributors, while in women, only Hb (coeff. β_Hb_: 0.176 + 0.085, *P*= 0.041) contributed at the borderline significance to the variability of ‘cumulative GPIIb/IIIa expression’.

**Table 6:** Multivariate regression parameters for testosterone and other co-explanatory variables for the model of ‘cumulative blood platelet reactivity’ as a dependent variable.

*variable*	*beta coefficient*	*std error of beta coefficient*	*-95%CI*	*+95%CI*	*determination coefficient [R^2^]*	*partial correlation*	*semipartial correlation*	*significance*
testosterone	-0.644	0.270	-1.179	-0.110	0.938	-0.194	-0.161	0.018
	^m^ -0.172	^m^ 0.087	^m^ -0.314	^m^ -0.030	^m^ 0.243	^m^ -0.165	^m^ -0.147	^m^ 0.043
	^f^ -0.223	^f^ 0.077	^f^ -0.406	^f^ -0.041	^f^ 0.165	^f^ -0.203	^f^ -0.172	^f^ 0.004
uric acid	-0.259	0.080	-0.417	-0.102	0.282	-0.261	-0.220	0.001
	^m^ -0.345	^m^ 0.078	^m^ -0.484	^m^ -0.206	^m^ 0.126	^m^ -0.352	^m^ -0.329	^m^ <<0.0001
	^f^ -0.212	^f^ 0.084	^f^ -0.381	^f^ -0.043	^f^ 0.306	^f^ -0.208	^f^ -0.177	^f^ 0.011
platelet count	0.257	0.076	0.108	0.407	0.203	0.272	0.230	0.001
	^m^ 0.271	^m^ 0.090	^m^ 0.105	^m^ 0.437	^m^ 0.335	^m^ 0.245	^m^ 0.221	^m^ 0.003
	^f^ 0.309	^f^ 0.074	^f^ 0.164	^f^ 0.454	^f^ 0.137	^f^ 0.313	^f^ 0.273	^f^ <<0.0001
glucose	0.232	0.077	0.081	0.384	0.223	0.244	0.205	0.003
	^m^ 0.285	^m^ 0.082	^m^ 0.123	^m^ 0.447	^m^ 0.199	^m^ 0.288	^m^ 0.263	^m^ 0.001
	^f^ 0.188	^f^ 0.082	^f^ 0.034	^f^ 0.342	^f^ 0.256	^f^ 0.188	^f^ 0.159	^f^ 0.023
homocysteine	0.200	0.074	0.053	0.347	0.175	0.218	0.182	0.008
	^m^ 0.240	^m^ 0.082	^m^ 0.080	^m^ 0.400	^m^ 0.214	^m^ 0.237	^m^ 0.214	^m^ 0.004
	^f^ 0.188	^f^ 0.074	^f^ 0.015	^f^ 0.362	^f^ 0.147	^f^ 0.208	^f^ 0.177	^f^ 0.013
age	-0.140	0.072	-0.282	0.002	0.117	-0.160	-0.131	0.053
	^m^ -0.088	^m^ 0.081	^m^ -0.223	^m^ 0.046	^m^ 0.191	^m^ -0.097	^m^ -0.085	^m^ 0.272
	^f^ -0.216	^f^ 0.073	^f^ -0.371	^f^ -0.061	^f^ 0.138	^f^ -0.245	^f^ -0.209	^f^ 0.003
cholesterol	-0.091	0.076	-0.242	0.060	0.219	-0.098	-0.080	0.237
	^m^ -0.161	^m^ 0.080	^m^ -0.297	^m^ -0.024	^m^ 0.159	^m^ -0.168	^m^ -0.149	^m^ 0.046
	^f^ -0.053	^f^ 0.076	^f^ -0.207	^f^ -0.102	^f^ 0.181	^f^ -0.082	^f^ -0.068	^f^ 0.488
leukocyte count	0.038	0.078	-0.116	0.191	0.243	0.040	0.033	0.627
	^m^ -0.082	^m^ 0.096	^m^ -0.254	^m^ 0.090	^m^ 0.413	^m^ -0.068	^m^ -0.060	^m^ 0.389
	^f^ 0.111	^f^ 0.079	^f^ -0.017	^f^ 0.239	^f^ 0.220	^f^ 0.124	^f^ 0.103	^f^ 0.162
haemoglobin	0.033	0.088	-0.141	0.208	0.417	0.031	0.026	0.706
	^m^ 0.094	^m^ 0.091	^m^ -0.057	^m^ 0.245	^m^ 0.336	^m^ 0.090	^m^ 0.079	^m^ 0.295
	^f^ 0.002	^f^ 0.073	^f^ -0.144	^f^ 0.148	^f^ 0.120	^f^ -0.014	^f^ -0.011	^f^ 0.983
sex	0.320	0.274	-0.222	0.862	0.939	0.096	0.079	0.245
								

The coefficients presented as the bootstrap-boosted standardized beta coefficients and their standard errors; n = 155. The dependent variable (‘cumulative blood platelet reactivity’) contained the van der Waerden normal scores of A_max_ cumulated through the agonists used (AA, collagen, ADP). *Per analogiam*, the bootstrap-boosted multiple regression models, including the same variables, were iterated separately for men (^m^) and for women (^f^) by means of the bootstrap resampling with replacement adjusted for sample size of the complete group (n =155) (10000 iterations). The multiple correlation coefficient (R) and the corrected overall determination coefficient (R^2^*_corr_*) for the model were: R = 0.582 and R^2^*_corr_* = 0.294, *P*<< 0.0001, for the overall group (n=155); R = 0.484 and (R^2^*_corr_*) = 0.187, *P*<< 0.0001, for men (n*_resampled_* =155); R = 0.558 and (R^2^*_corr_*) = 0.269, *P*<< 0.0001, for women (n*_resampled_* =155).

Multivariate regression parameters for dihydrotestosterone and co-explanatory variables for the model of ‘cumulative blood platelet reactivity’ as a dependent variable are given in Table 7. In the model with DHT, the significant variables in the model were platelet count, uric acid, glucose, Hcy and DHT. DHT demonstrated the highest absolute value of the standardized beta coefficient, which implies that it contributed to explaining the variability of the dependent variable (‘cumulative blood platelet reactivity) to the greatest extent. Both the partial correlation coefficient and semipartial correlation coefficient were moderate (*P*< 0.02), which suggests that the contribution of DHT was not dominating over other independent variables in the model (the univariate correlation coefficient between DHT and ‘cumulative blood platelet reactivity’ *r*_P_= -0.393, *P*<< 0.0001). In the model with DHT, the multiple regression analysis performed separately for sexes revealed that in men, uric acid, glucose, platelet count, Hcy and DHT were the most important contributors to the variability of the ‘cumulative blood platelet reactivity’; in addition, uric acid, glucose, platelet count and Hcy demonstrated the most significant partial and semipartial correlations, indicating that they had the greatest individual impact in the model. In a separate group of women, it was found that platelet count, DHT and uric acid appeared the most important contributors to the ‘cumulative blood platelet reactivity’ variability, while platelet count and DHT demonstrated the highest individual impact in the model (Table 7, the data with the superscript ^m^ and ^f^).

**Table 7:** Multivariate regression parameters for dihydrotestosterone and other co-explanatory variables for the model of ‘cumulative blood platelet reactivity’ as a dependent variable.

*variable*	*beta coefficient*	*std error of beta coefficient*	*-95%CI*	*+95%CI*	*determination coefficient [R^2^]*	*partial correlation*	*semipartial correlation*	*significance*
dihydrotestosterone	-0.602	0.206	-1.009	-0.196	0.894	-0.236	-0.196	0.004
	^m^ -0.182	^m^ 0.084	^m^ -0.282	^m^ -0.081	^m^ 0.183	^m^ -0.191	^m^ -0.170	^m^ 0.026
	^f^ -0.247	^f^ 0.077	^f^ -0.399	^f^ -0.095	^f^ 0.168	^f^ -0.227	^f^ -0.192	^f^ 0.001
platelet count	0.262	0.075	0.114	0.410	0.203	0.279	0.234	0.001
	^m^ 0.274	^m^ 0.090	^m^ 0.105	^m^ 0.443	^m^ 0.339	^m^ 0.251	^m^ 0.227	^m^ 0.002
	^f^ 0.303	^f^ 0.073	^f^ 0.154	^f^ 0.452	^f^ 0.138	^f^ 0.324	^f^ 0.281	^f^ <<0.0001
uric acid	-0.259	0.079	-0.414	-0.103	0.277	-0.264	-0.220	0.001
	^m^ -0.357	^m^ 0.078	^m^ -0.499	^m^ -0.215	^m^ 0.128	^m^ -0.355	^m^ -0.332	^m^ <<0.0001
	^f^ -0.203	^f^ 0.083	^f^ -0.368	^f^ -0.038	^f^ 0.299	^f^ -0.193	^f^ -0.162	^f^ 0.014
glucose	0.236	0.075	0.087	0.385	0.214	0.252	0.210	0.002
	^m^ 0.280	^m^ 0.080	^m^ 0.118	^m^ 0.443	^m^ 0.180	^m^ 0.293	^m^ 0.268	^m^ <<0.0001
	^f^ 0.186	^f^ 0.082	^f^ 0.032	^f^ 0.341	^f^ 0.300	^f^ 0.182	^f^ 0.152	^f^ 0.024
homocysteine	0.195	0.074	0.050	0.341	0.173	0.215	0.178	0.009
	^m^ 0.232	^m^ 0.081	^m^ 0.057	^m^ 0.406	^m^ 0.203	^m^ 0.236	^m^ 0.212	^m^ 0.005
	^f^ 0.190	^f^ 0.074	^f^ 0.007	^f^ 0.374	^f^ 0.169	^f^ 0.200	^f^ 0.168	^f^ 0.011
age	-0.136	0.071	-0.277	0.005	0.118	-0.157	-0.128	0.058
	^m^ -0.100	^m^ 0.080	^m^ -0.230	^m^ 0.031	^m^ 0.185	^m^ -0.110	^m^ -0.096	^m^ 0.210
	^f^ -0.192	^f^ 0.073	^f^ -0.354	^f^ -0.031	^f^ 0.133	^f^ -0.217	^f^ -0.182	^f^ 0.009
total cholesterol	-0.075	0.076	-0.225	0.074	0.222	-0.082	-0.067	0.321
	^m^ -0.157	^m^ 0.079	^m^ -0.295	^m^ -0.020	^m^ 0.149	^m^ -0.161	^m^ -0.142	^m^ 0.048
	^f^ -0.037	^f^ 0.076	^f^ -0.199	^f^ 0.125	^f^ 0.202	^f^ -0.065	^f^ -0.053	^f^ 0.619
haemoglobin	0.022	0.088	-0.151	0.196	0.420	0.021	0.017	0.802
	m 0.062	m 0.087	m -0.095	m 0.218	m 0.295	m 0.057	m 0.050	m 0.468
	^f^ 0.019	^f^ 0.072	^f^ -0.122	^f^ 0.159	^f^ 0.115	^f^ 0.017	^f^ 0.014	^f^ 0.792
leukocyte count	0.018	0.078	-0.135	0.172	0.258	0.020	0.016	0.812
	^m^ -0.067	^m^ 0.093	^m^ -0.236	^m^ 0.102	^m^ 0.382	^m^ -0.063	^m^ -0.055	^m^ 0.469
	^f^ 0.071	^f^ 0.081	^f^ -0.063	^f^ 0.204	^f^ 0.278	^f^ 0.073	^f^ 0.060	^f^ 0.381
sex	0.277	0.216	-0.149	0.704	0.904	0.106	0.086	0.201
								

The coefficients are presented as the bootstrap-boosted standardized beta and their standard errors; n = 155. The dependent variable (‘cumulative blood platelet reactivity’) contained the van der Waerden normal scores of A_max_ cumulated through the agonists used (AA, collagen, ADP). *Per analogiam*, the bootstrap-boosted multiple regression models including the same variables were iterated separately for men (^m^) and for women (^f^) by bootstrap resampling with replacement adjusted for the sample size of the complete group (n=155) (10000 iterations). The multiple correlation coefficient (R) and the corrected overall determination coefficient (R^2^*_corr_*) for the model were: R =0.593 and R^2^*_corr_* =0.307, *P*<< 0.0001, for the overall group (n=155); R =0.488 and (R^2^*_corr_*) =0.191, *P*<< 0.0001, for men (n*_resampled_* =155); R =0.570 and (R^2^*_corr_*) =0.283, *P*<< 0.0001, for women (n*_resampled_* =155).

For the ‘cumulative platelet activation’, used as a dependent variable, only haemoglobin and leukocyte count appeared to be significant independent variables (resp., coeff. β: 0.237 + 0.107, *P*< 0.03 and coeff. β: -0.189 + 0.095, *P*< 0.05), while DHT remained beyond significance (coeff. β: -0.453 + 0.273, *P*= 0.099). In a separate subgroup of men, only glucose contributed significantly to the overall variability of the model (coeff. β: -0.324 + 0.085, *P*= 0.0002), while in the subgroup of women, it was Hcy and DHT (coeff. β_Hcy_: -0.283 + 0.082, *P*< 0.001 and coeff. β_DHT_: -0.172 + 0.085, *P*< 0.05).

When the van der Waerden normal scores of the ‘cumulative GPIIb/IIIa expression’ were employed as a dependent variable, Hcy remained the only significant independent variable (coeff. β: -0.183 + 0.090, *P*< 0.05), while DHT was beyond significance (coeff. β: -0.516 + 0.272, *P*= 0.059). In men, Hcy and glucose contributed significantly to the variability of ‘cumulative GPIIb/IIIa expression’ (coeff. β_Hcy_: -0.327 + 0.087, *P*= 0.0002 and coeff. β_glucose_: -0.237 + 0.086, *P*= 0.006), whereas haemoglobin was the only significant independent variable in women (coeff. β: 0.170 + 0.082, *P*= 0.042). To sum up this part, both T and DHT contribute significantly to blood platelet reactivity, both in the overall group of patients and in separate subgroups of men and women. However, it is important to note that the extent of such a contribution is strongly affected by confounding factors. The concentrations of both androgens are not, however, significant predictors of platelet activation or expression of P-selectin or GPIIb/IIIa on blood platelets.

A logistic regression analysis was performed to determine how selected analysed (confounding/co-explanatory) variables contribute to lower or higher blood platelet reactivity cumulated through the used agonists (AA, collagen and ADP in the case of aggregometry, AA and collagen in the case of flow cytometry). The dependent variables were the dichotomised values of the variables referred to as ‘cumulative blood platelet reactivity’, the dichotomised values of the variables referred to as ‘cumulative platelet activation’ or ‘cumulative P-selectin/GPIIb/IIIa expression’.

In the whole group, both T and DHT, adjusted for sex and age, appeared as significant predictors of the ‘cumulative blood platelet reactivity’ (OR_T_ = 1.002*10^-10^ [95%CI: 4.956*10^-19^ – 0.202*10^-1^, *P*< 0.02] and OR_DHT_ = 6.842*10^-12^ [95%CI: 1.244*10^-19^ – 0.376*10^-3^, *P*< 0.005]). Sex- and age-adjusted T also remained a significant predictor when standardized individually for blood haemoglobin or leukocyte count (for both *P*< 0.02), platelet count (*P*< 0.005), mean platelet volume (*P*< 0.025) or plateletcrit (*P*< 0.01), total or HDL-cholesterol (for both *P*< 0.02), glucose (*P*< 0.01) or Hcy level (*P*< 0.02), but not upon standardization for uric acid (*P*= 0.061). Upon the overall multiple *post hoc* standardization for platelet and leukocyte counts, haemoglobin, cholesterol, Hcy, glucose and uric acid the resultant multivariate OR_T,_ *_multivar_* was 3.618*10^-11^ [95%CI: 1.145*10^-20^ – 0.114, *P*< 0.03] (*P*_Hosmer-Lemeshow_= 0.538). When adjusted for gender and age, DHT remained a significant predictor of the dichotomised ‘cumulative blood platelet reactivity’ upon its *post hoc* individual standardization for blood platelet count or plateletcrit (for both *P*< 0.0005), haemoglobin (*P*< 0.04), total or HDL-cholesterol (for both *P*< 0.01), glucose (*P*< 0.01), uric acid (*P*< 0.02) or Hcy (*P*< 0.005), but not upon standardization for leukocyte count (*P*= 0.051). Upon *post hoc* multiple standardization for platelet and leukocyte counts, haemoglobin, cholesterol, homocysteine and uric acid, the overall age- and sex-adjusted multivariate OR was OR_DHT,_ *_multivar_* = 1.318*10^-13^ [95%CI: 2.270*10^-23^ – 0.627*10^-3^, *P*< 0.01] (*P*_Hosmer-Lemeshow_= 0.744). Thus, concentrations of T and DHT appear to be significant predictors of lower platelet aggregability, and remained so upon adjustment of the models, not only for sex and age, but also for several other studied variables.

Otherwise, only DHT adjusted for age appeared to be the significant predictor of the ‘cumulative blood platelet reactivity’ in separated subgroups of men (OR_DHT_ = 0.585*10^-10^ [95%CI: 0.536*10^-20^ – 0.638, *P*< 0.05]) and women (OR_DHT_ = 1.091*10^-12^ [95%CI: 1.106*10^-19^ – 1.076*10^-5^, *P*< 0.001]): no significant relationships were observed for T adjusted only for age, in neither men nor women. In men, age-adjusted DHT also maintained a significant impact when standardized individually for blood haemoglobin (*P*= 0.05), mean platelet volume and total, HDL- or LDL-cholesterol (for all *P*< 0.05), platelet count (*P*< 0.004), plateletcrit (*P*< 0.01), uric acid or Hcy (for both *P*< 0.04), but not upon standardization for leukocyte count (*P*= 0.094). In turn, in women, DHT maintained a significant impact also upon individual standardization for plateletcrit, leukocyte count, HDL-cholesterol or Hcy (for all *P*< 0.001), blood haemoglobin, mean platelet volume, total and LDL-cholesterol or glucose (for all *P*< 0.002), platelet count (*P*< 0.0005) and uric acid (*P*< 0.003). When the model with age-adjusted ‘cumulative blood platelet reactivity’, used as a dichotomous dependent variable, was subjected to *post hoc* multiple-standardization for a set of independent predictors including T or DHT, platelet and leukocyte counts, haemoglobin, cholesterol, homocysteine and uric acid level, the multivariate OR appeared particularly significant for DHT in both sexes (OR_DHT-men,_ *_multivar_* = 1.002*10^-22^ [95%CI: 9.423*10^-37^ – 1.065*10^-8^, *P*= 0.002]; *P*_Hosmer-Lemeshow_= 0.123 and OR_DHT-women,_ *_multivar_* = 4.472*10^-17^ [95%CI: 1.864*10^-27^ – 1.072*10^-6^, *P*= 0.01]; *P*_Hosmer-Lemeshow_= 0.731), and less significant for T (OR_T-men,_ *_multivar_* = 2.374*10^-15^ [95%CI: 1.926*10^-27^ – 2.926*10^-3^, *P*= 0.018]; *P*_Hosmer-Lemeshow_= 0.195 OR_T-women,_ *_multivar_* = 3.809*10^-17^ [95%CI: 3.789*10^-27^ – 3.830*10^-7^, *P=* 0.001]; *P*_Hosmer-Lemeshow_= 0.063).

For the ‘cumulative platelet activation’, the model with DHT as an explanatory variable, adjusted for age and gender and subjected to *post hoc* normalisation for additional confounders, appeared at the borderline of significance for individually-included confounders (*P*> 0.08). The multivariate model with sex, age, platelet and leukocyte counts, haemoglobin, total cholesterol, glucose, uric acid and Hcy also remained beyond statistical significance (*P*= 0.078) (*P*_Hosmer-Lemeshow_= 0.619). Otherwise, when T was chosen as an explanatory variable in the model, adjusted for age and gender and subjected to *post hoc* normalisation for additional confounders, the significance was even poorer for the models with individual confounders and for the multivariate model (*P*= 0.089) (*P*_Hosmer-Lemeshow_= 0.404).

For the ‘cumulative GPIIb/IIIa expression’ the models with testosterone or DHT as the explanatory variables, adjusted for age and gender and subjected to *post hoc* normalisation for additional confounders, were significant neither for individually-included confounders nor for the multivariate model comprising sex, age, platelet and leukocyte counts, haemoglobin, total cholesterol, glucose, uric acid and Hcy (*P*> 0.1) (*P*_Hosmer-Lemeshow_= 0.163 and *P*_Hosmer-Lemeshow_= 0.202 for T and DHT, resp.).

Finally, a linear discriminant analysis (LDA) was performed to determine which analysed confounding/co-explanatory variables contribute the most to the discrimination between lower and higher blood platelet reactivity (dichotomised values of ‘cumulative blood platelet reactivity’, ‘cumulative platelet activation’ and ‘cumulative GPIIb/IIIa expression’ used as grouping variables) depending on sex. In general, based on the values of partial Wilk’s lambda estimated for all patients together (^m^λ_partial Wilks_ and ^f^λ_partial Wilks_ denote the respective values of partial Wilk’s lambda for separate subgroups of men and women adjusted for the sample size of *n*= 155), the most discriminative variables for dichotomised A_max_ cumulated through agonists (‘cumulative blood platelet reactivity’), in the standard LDA model appear to be platelet count (λ_partial Wilks, plt_ =0.912, *P*< 0.0003; ^m^λ_partial Wilks, plt_ =0.896, *P*< 0.0002; ^f^λ_partial Wilks, plt_ =0.869, *P*<< 0.0001), uric acid (λ_partial Wilks, uric acid_ = 0.924, *P*<0.001; ^m^λ_partial Wilks, uric acid_ =0.925, *P*< 0.002; ^f^λ_partial Wilks, uric acid_ =0.936, *P*< 0.002), DHT (λ_partial Wilks, DHT_ = 0.884, *P*<< 0.0001; ^m^λ_partial Wilks, DHT_ =0.899, *P*< 0.0002; ^f^λ_partial Wilks, DHT_ =0.908, *P*< 0.0002), T (λ_partial Wilks, T_ = 0.907, *P*< 0.002; ^m^λ_partial Wilks, T_ =0.946, *P*< 0.01; ^f^λ_partial Wilks, T_ =0.945, *P*< 0.003) and glucose (λ_partial Wilks, glucose_ = 0.971, *P*< 0.03; ^m^λ_partial Wilks, glucose_ =0.925, *P*< 0.002; ^f^λ_partial Wilks, glucose_ =0.980, *P*= 0.076). However, the only significant variables identified in the model using the backward stepwise approach were platelet count (λ_partial Wilks, plt_ = 0.895, *P*<< 0.0001; ^m^λ_partial Wilks, plt_ =0.871, *P*<< 0.0001; ^f^λ_partial Wilks, plt_ =0.842, *P*<< 0.0001), DHT (λ_partial Wilks, DHT_ =0.853, *P*<< 0.0001; ^m^λ_partial Wilks, DHT_ =0.919, *P*< 0.001; ^f^λ_partial Wilks, DHT_ =0.903, *P*< 0.0001) and T (λ_partial Wilks, T_ =0.877, *P*<< 0.0001; ^m^λ_partial Wilks, T_ =0.962, *P*< 0.02; ^f^λ_partial Wilks, T_ =0.907, *P*< 0.0001). For men DHT (λ_partial Wilks_= 0.893, *P*<< 0.0001), platelet count (λ_partial Wilks_= 0.911, *P*< 0.0005) and uric acid (λ_partial Wilks_= 0.961, *P*< 0.02) appeared the most discriminative variables for the same grouping variable (dichotomised ‘cumulative blood platelet reactivity’), whereas also DHT (λ_partial Wilks_ =0.859, *P*<< 0.0001) and platelet count (λ_partial Wilks_= 0.897, *P*<< 0.0001) discriminated the best in the backward stepwise approach. In women, platelet count and uric acid most clearly discriminated (with λ_partial Wilks_= 0.911, *P*< 0.015 and λ_partial Wilks_= 0.946, *P*< 0.05) for the same grouping variable (dichotomised ‘cumulative blood platelet reactivity’).

For the ‘cumulative platelet activation’ as the grouping variable, upon adjustment for sex and age, T and DHT remained the best discriminators (with λ_partial Wilks_= 0.970, *P*< 0.04 and λ_partial Wilks_= 0.965, *P*< 0.03 for separate models; stepwise forward analyses). When the expression of P-selectin (cumulated through agonists) was used as a grouping variable, testosterone and DHT), adjusted for sex and age, also appeared the most significant discriminators (with λ_partial Wilks_= 0.960, *P*< 0.02 and λ_partial Wilks_= 0.958, *P*< 0.02, respectively; stepwise forward approach).

Overall, both tested androgens, DHT and T appeared to significantly discriminate platelet ‘lower’ and ‘higher’ reactivity in the overall group including both sexes, however, the discriminative potential of T was markedly less expressed, particularly in sex subgroups. The androgens also remained significant predictors of platelet surface membrane GPIIb/IIIa expression. In the whole group of patients (incl. both males and females), as well as in the separated sex subgroups, platelet count, DHT/T and uric acid appeared the leading modulators of platelet reactivity.

### *In vitro* effects of sex steroids on whole blood platelet aggregation

All the tested hormones demonstrated *in vitro* inhibitory effects on agonist-stimulated platelet aggregation, even at very low steroid concentrations. In general, sex was not found to have any significant effect when analysing the inhibitions of platelet aggregation by T, DHT and E_2_ in separate groups of men and women.

**Figure 1:**
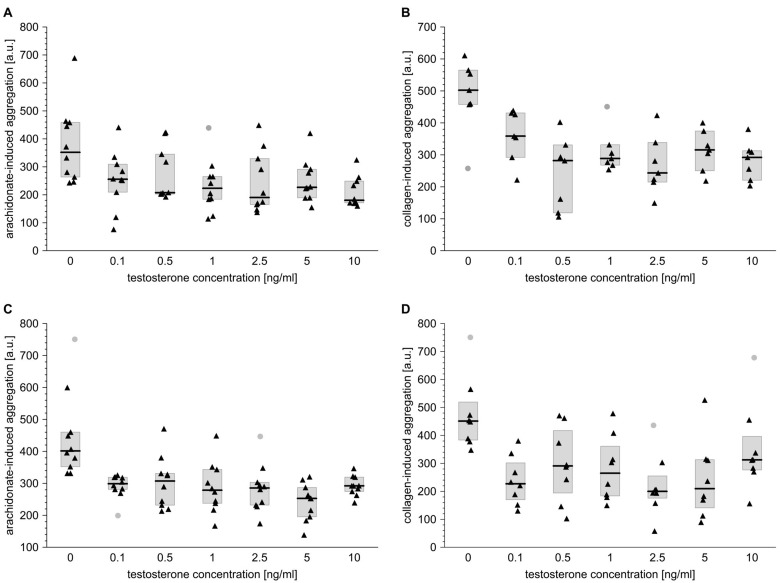
**Testosterone affects platelet aggregation induced by arachidonate or collagen in blood taken from men and from women.** Data is presented as medians (thick horizontal lines) and interquartile ranges (IQR) (boxes, from lower quartile [25%] to upper quartile [75%]). Raw data is presented as black solid triangles or grey solid circles (outliers, by two-sided Tukey’s test: 1.5*[IQR]) for whole blood platelets stimulated with either arachidonate (0.5 mmol/l) (**A**, **C**) or collagen (1 µg/ml) (**B**, **D**) in men (**A, B**) and women (**C, D**). For experimental details, see *Materials and methods*. The significance of differences was estimated for Box-Cox-transformed data by the bootstrap-boosted (10000 iterations) ANOVA for repeated measures and the paired Student’s t-test with Bonferroni’s correction for *post hoc* multiple comparisons: *P* < 0.0005, µ_0_ ≠ µ_0.1_ = µ_0.5_ = µ_1_ = µ_2.5_ = µ_5_ = µ_10_ for arachidonate-activated platelets in men; *P* < 0.001, µ_0_ ≠ µ_0.1_ = µ_0.5_ = µ_1_ = µ_2.5_ = µ_5_ = µ_10_ for collagen-activated platelets in men; *P* < 0.001, µ_0_ ≠ µ_0.1_ = µ_0.5_ = µ_1_ = µ_2.5_ = µ_5_ = µ_10_ for arachidonate-activated platelets in women; *P* < 0.05, µ_0_ ≠ µ_0.1_ = µ_0.5_ = µ_1_ = µ_2.5_ = µ_5_ = µ_10_ for collagen-activated platelets in women.

Briefly, T appeared to be a potent inhibitor of 1 µg/ml collagen-dependent aggregation of blood platelets in both men and women. After the addition of T at final concentrations of 0.1, 0.5, 1, 2.5, 5 or 10 ng/ml, respective significant inhibition of platelet aggregation by 30, 45, 44, 52, 38 and 42% was observed in men (Fig. 1B), and by 50, 36, 41, 56, 54 and 31% in women (Fig. 1D), compared to control vehicle-treated samples.

Likewise, T effectively attenuated the extent of aggregation of male and female platelets induced with 0.5 mmol/l arachidonic acid: it reduced arachidonate-dependent platelet aggregation by 27, 41, 36, 45, 35 and 49%, respectively, in men (Fig. 1A) and by 26, 23, 31, 29, 37 and 27%, respectively, in women (Fig. 1C). Characteristically, the degree of T-induced anti-aggregatory effect was very similar regardless of T concentration.

**Figure 2:**
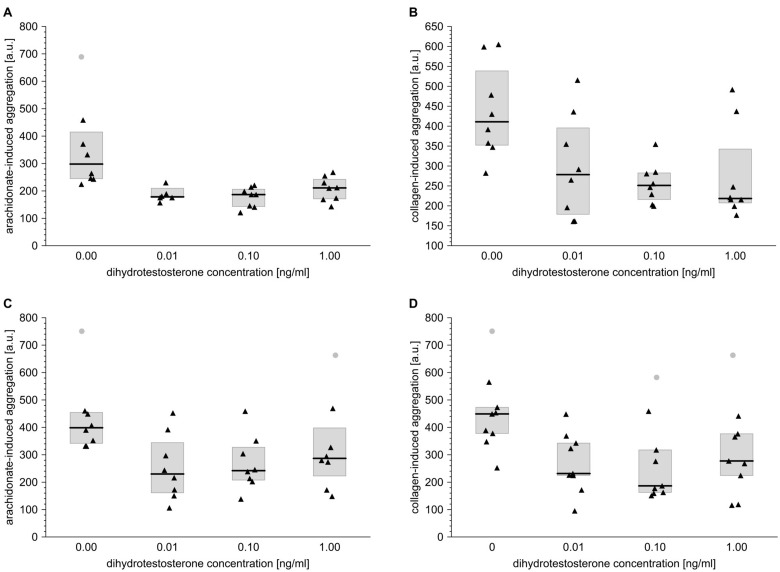
**Dihydrotestosterone affects platelet aggregation induced by arachidonate or collagen in blood taken from men and from women.** Data is presented as medians (thick horizontal lines) and interquartile ranges (IQR) (boxes, from lower quartile [25%] to upper quartile [75%]). Raw data is presented as black solid triangles or grey solid circles (outliers, by two-sided Tukey’s test: 1.5*[IQR]) for whole blood platelets stimulated with either arachidonate (0.5 mmol/l) (**A**, **C**) or collagen (1 µg/ml) (**B**, **D**) in men (**A**, **B**) and women (**C**, **D**). For experimental details see *Materials and methods*. The significance of differences was estimated for Box-Cox-transformed data by the bootstrap-boosted (10000 iterations) ANOVA for repeated measures and the paired Student’s t test with Bonferroni’s correction for *post hoc* multiple comparisons: *P* < 0.001, µ_0_ ≠ µ_0.01_ = µ_0.1_ = µ_1_ for arachidonate-activated platelets in men; *P* < 0.01, µ_0_ ≠ µ_0.01_ = µ_0.1_ = µ_1_ for collagen-activated platelets in men; *P* < 0.01, µ_0_ ≠ µ_0.01_ = µ_0.1_ = µ_1_ for arachidonate-activated platelets in women; *P* < 0.01, µ_0_ ≠ µ_0.01_ = µ_0.1_ for collagen-activated platelets in women.

When blood obtained from men or women was incubated with DHT, significant anti-aggregatory effects were observed in the case of collagen-dependent platelet reactivity. DHT at concentrations of 0.01, 0.1 and 1 ng/ml inhibited aggregation to similar extents, *i.e.* by 32, 39 and 47%, respectively, in men (Fig. 2B) and by 48, 59 and 38%, respectively, in women (Fig. 2D).

Also, in the case of arachidonate-induced aggregation, DHT acted as an efficient inhibitor in blood samples taken from men (inhibition by 40, 35 and 29%, resp. for the increasing DHT concentrations) or women (inhibition by 39, 40 and 30%, resp. for the increasing DHT concentrations) (Fig. 2A, C).

**Figure 3:**
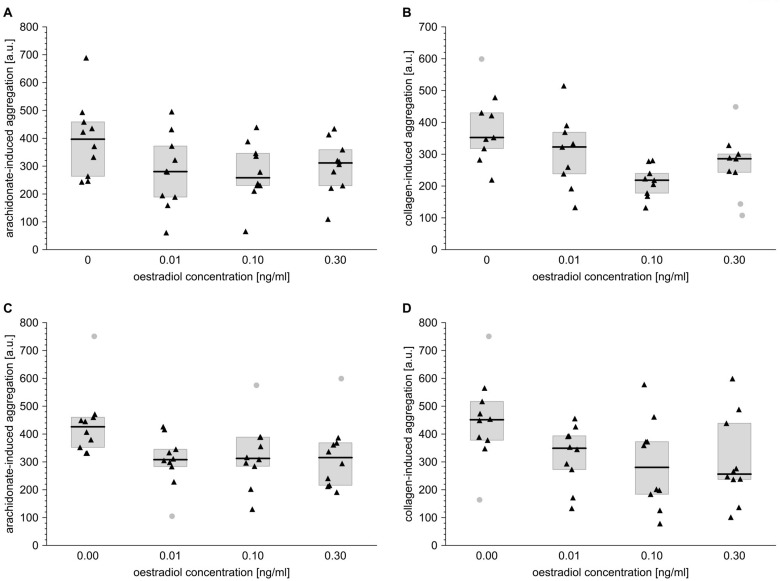
**Oestradiol affects platelet aggregation induced by arachidonate or collagen in male and female blood.** Data s presented as medians (thick horizontal lines) and interquartile ranges (IQR) (boxes, from lower quartile [25%] to upper quartile [75%]). Raw data is presented as black solid triangles or grey solid circles (outliers, by two-sided Tukey’s test: 1.5*[IQR]) for whole blood platelets stimulated with either arachidonate (0.5 mmol/l) (**A**, **C**) or collagen (1 µg/ml) (**B**, **D**) in men (**A**, **B**) and women (**C**, **D**). For experimental details, see *Materials and methods*. The significance of differences is estimated for Box-Cox-transformed data by the bootstrap-boosted (10000 iterations) ANOVA for repeated measures and the paired Student’s t-test with Bonferroni’s correction for *post hoc* multiple comparisons: *P* < 0.01, µ_0_ ≠ µ_0.01_ = µ_0.1_ = µ_0.3_ for arachidonate-activated platelets in men; *P* < 0.01, µ_0_ ≠ µ_0.1_ = µ_0.3_ for collagen-activated platelets in men; *P* < 0.01, µ_0_ ≠ µ_0.1_ = µ_0.3_ for arachidonate-activated platelets in women; *P* < 0.05, µ_0_ ≠ µ_0.01_ = µ_0.1_ = µ_0.3_ for collagen-activated platelets in women.

E_2_ inhibited platelet aggregation induced by either collagen or AA. When used at increasing concentrations (0.01, 0.1 and 0.3 ng/ml), E_2_ significantly inhibited collagen-triggered platelet aggregation by 8, 38 and 19% in men (Fig. 3B) and by 23, 38 and 43% in women (Fig. 3D). Also, according to our *in vitro* aggregatory measurements, E_2_ exhibited significant antiplatelet properties in the blood of both male and female subjects, when arachidonate was used to trigger platelet aggregation. E_2_ used at final concentrations of 0.01, 0.1 or 0.3 ng/ml inhibited arachidonic acid-induced platelet aggregation by 29, 35 and 21% in men and by 28, 27 26% in women (Fig. 3A, C).

## DISCUSSION

Our results clearly show that T and DHT possess anti-platelet potential. Outcomes suggesting such a conclusion are derived not only from the *in vivo/ex vivo* findings presented in this study, but also from an *in vitro* model evaluating the effects of exogenous steroids on platelet reactivity. To ensure that the identified relationships remained valid upon adjustment for several confounders that are very likely to shape the extent of blood platelet reactivity, the *in vivo* findings were analysed using both simple association methods, without adjusting for numerous potential confounders, and multivariate approaches.

No concentration dependence was identified in the *in vitro* part of the aggregation study: similar extents of anti-aggregatory activity were found to occur for any of the tested concentrations of T and DHT. In the case of T, potent antiplatelet action was exerted even at concentrations corresponding to hypogonadal levels. These anti-aggregatory effects of androgens appeared sex-independent: the anti-aggregatory potential of T and DHT was revealed in blood samples originating from both men and women.

Our results regarding the *in vitro* effects of androgens on the aggregation of platelets stimulated with arachidonate or collagen confirm those of previous reports, showing that T has a significant inhibitory action on platelet aggregation induced by ADP, both in the presence and absence of endothelial cells [15]. Hence, our present findings expand the knowledge gathered to date, as previous studies have only examined the effects of T on ADP-dependent platelet aggregation [15]. The results revealed in the present paper suggest that T has a universal antiplatelet action, regardless of the agonist used to trigger platelet aggregation. Interestingly, the anti-aggregatory action may not be exceptional for androgens, since also E_2_ was found to have a strong anti-aggregatory effect under *in vitro* conditions, regardless of the used agonist (arachidonate or collagen) and regardless of the sex of blood donors.

Based on the outcomes presented by Campelo et al. [15] we can speculate that the inverse association between T/DHT plasma concentration and platelet reactivity, noted in the *ex vivo* part of this study, may be attributed to NO secreted from endothelial cells lining the vascular bed of the investigated volunteers. Nevertheless, the outcomes of our simplified *in vitro* model, focused on the effects of sex hormones on agonist-mediated whole blood platelet aggregation, may also lead to another hypothesis. The examined steroids were found to have distinct inhibitory effects, despite the absence of any cells from vascular wall. It is possible then that T and DHT can directly affect the functional state of blood platelets without the involvement of the endothelium. However, since blood platelets cooperate widely with other blood cells, it cannot be excluded that the observed *in vitro* and *in vivo* anti-aggregatory action of androgens in whole blood depends on cellular or humoral interactions with leukocytes and/or erythrocytes, both commonly known as important modulators of platelet aggregation [16,17].

The lack of a cell nucleus in blood platelets does not exclude the possibility of the direct action of steroids on blood platelets that are able to respond to T and DHT *via* a receptor-dependent or independent non-genomic route [18]. Due to their specific cellular structure, blood platelets appear as suitable model objects for studying the non-genomic effects of steroids. Blood platelets demonstrate quite fast responses to steroidal stimuli, previously observed following incubations lasting only a few to several minutes [15], and these findings may additionally suggest that the observed reactions are most likely genome-independent. Nevertheless, the exact mechanisms underlying the antiplatelet action of T and DHT, particularly with regard to the distinction between direct interaction with blood platelets or mediation by other components of vascular wall and blood cells, remain to be elucidated.

Regarding the platelet effects of T, a small body of evidence already exists concerning the relationship between androgens and primary haemostasis. However, our finding that the reduced form of T is able to modulate platelet activation and reactivity is a key novel aspect of the present study. Our results are in agreement with the those reported by Li et al., who observed a significant anti-aggregatory action of DHT under oxidative stress [19]. Both our present results and those reported by Li and co-workers [19] consistently indicate that DHT can reduce platelet reactivity both under basal conditions and under hydrogen peroxide-induced oxidative stress in response to arachidonate, collagen and ADP. However, there is too little evidence to unambiguously state that DHT works in a very similar way to T. More studies are needed to determine whether, and to what extent, this assumption remains correct.

In some of our models, DHT was even revealed as a more significant contributor to platelet reactivity than T. This is the first indication that DHT may have such an influence on the direct and indirect modulation of platelet haemostasis, and it clearly shows that not only T, but also its reduced form, plays an important role in atherogenesis, both in men and women.

In addition to our aggregation data, our results also demonstrate that T and DHT levels are negatively associated with the expression of the activated GPIIb/IIIa complex (the receptor for fibrinogen) in older men and women. The binding of fibrinogen by blood platelets is a crucial step on the path to haemostatic plug formation [20]. Regrettably, our present findings do not offer sufficient evidence to speculate whether the negative association between T and DHT levels and the expression of the activated form of GPIIb/IIIa complex occurs due to fewer copies of the activated fibrinogen receptor being present on platelet membranes, or whether it may be rather associated with structural alterations in the GPIIb/IIIa complex binding pocket, thus hampering fibrinogen binding.

Our results strongly suggest that androgens are negative regulators of platelet function, and hence that men may be better protected against the excessive activation and/or priming of circulating platelets. When using overall platelet reactivity as a cumulative marker, women were characterized by higher platelet reactivity and - as a consequence, dominated in the group showing ‘higher platelet reactivity’. In contrast, the group referred to as ‘lower platelet reactivity’ subjects was dominated by men. This discrimination further supports the hypothesis that androgens, which are present at higher concentrations in men, are hormones with antiplatelet activity. This trend remains even after adjusting the variables describing platelet reactivity for age and sex.

Our findings indicate that androgens concentrations correlate negatively with various indices of platelet morphology: higher levels of androgens correspond to lower MPV, PCT, PDW and P-LCR. Since women possess lower levels of androgens, they also demonstrate lower levels of hormonal factors, which contribute to reduced MPV, PCT, PDW and P-LCR levels. Accordingly, they present higher values of platelet morphology indices, compared to men.

It is well known that the variables like platelet count, platelet volume and a number of large platelets reflect the rate of thrombopoiesis: faster production of new platelets are assumed to be associated with higher platelet counts, higher MPV and higher P-LCR values in blood morphology analysis. Higher values of platelet morphology indices, especially MPV and PDW, are supposed to predict higher platelet reactivity [21,22]. Indeed, our findings confirm significant positive associations between platelet reactivity and MPV, PLT, PCT and P-LCR, not only for the group as a whole, but also in separate sex subgroups. One might speculate that faster thrombopoiesis may be associated with lower androgen levels and that androgens negatively modulate the rate of thrombopoiesis, thus contributing to lower platelet reactivity. As thrombopoiesis was not monitored directly in the present study, , any relationships of this type are rather speculative and remain to be further explored.

Our present findings show that glucose, cholesterol and Hcy are significantly associated with plasma levels of T and DHT. Noteworthy, however, merely for glucose, but not for cholesterol or Hcy, such associations are maintained regardless of whether analysed in a whole group or in separate sex subgroups of elderly individuals. All of the above variables are known to strongly affect platelet activation and reactivity [23–25]. Therefore, poorly-recognized pathways dependent on cholesterol, glucose or Hcy, which orchestrate platelet activation and reactivity, may serve as either mediators or effectors of the indirect haemostatic action of androgens, in addition to their modulation of the concentrations of endothelium-derived NO.

The level of Hcy appears to positively correlate with T and DHT. Hcy is a sulphur amino acid which acts a strong enhancer of platelet reactivity and activation; it also modulates the sensitivity of platelets to the inhibitory action of acetylsalicylic acid [25,26]. Physiological levels of homocysteinaemia in plasma are believed to be higher in men than in women [27], as confirmed by our results, which show significantly increased plasma homocysteinaemia in men. We propose that T is the biochemical mediator of this relationship.

The most likely molecular basis for the “male sex – homocysteine – testosterone” triad is the T-dependent regulation of the activity of cystathionine β-synthase (CBS) taking place in the human kidney [28]. CBS catalyzes the conversion of Hcy to cystathionine, and when the rate of this conversion slows due to decreased CBS activity induced by T, Hcy concentration increases. Interestingly, higher homocysteinaemia has been associated with the use of some analogues of T: for example, anabolic-androgenic steroids, such as methanedienone, oxymethalone, stanazolol, methanolone enenthate, boldenone and trenbolone [29]. Therefore, it can be suggested that androgens are positive regulators of Hcy levels *via* their inhibitory impact on CBS activity, and hence they may indirectly affect platelet sensitivity to activators and inhibitors. Whether DHT really affects plasma concentrations of Hcy in the manner similar to T is a matter of further debate, and our present data is the first to indicate such a possible association between DHT and Hcy.

In addition, our findings indicate a significant positive association between T/DHT levels and uric acid concentration in plasma. Concentrations of uric acid are also higher in men than women, suggesting that sex-specific differences in androgen concentrations may account for the relationship between sex and uricaemia. An inverse association between uric acid and platelet aggregability was noted in both sexes, and this may suggest that uric acid is an endogenous antiplatelet compound. Hence, uric acid appears to act as an anti-atherogenic compound. Interestingly, this finding clearly contradicts some earlier reports suggesting that uric acid exhibits proatherogenic action, and may be used to predict life-threatening cardiovascular events [30]. However, according to the findings presented herein, androgens seem to elevate plasma levels of uric acid, which in turn, decreases the extent of platelet aggregation. Therefore, the true basis for the negative association between androgens and platelet aggregability would be the “androgens – uric acid – platelets” pathway.

Such a hypothesis, however, although interesting, needs further detailed verification. In fact, exogenous T has already been described as an enhancer of uric acid concentration in women treated with testosterone replacement therapy for gender identity disorders [31]. Our results are in line with those obtained by Kurahashi and co-workers, but significantly, our findings also show that not only endogenous T, but also endogenous DHT, acts as a physiological positive regulator of uric acid concentration in subjects of both sexes, even those not subjected to any hormonal replacement therapies.

The molecular background, associated with a positive correlation between concentration of T and the level of uric acid, refers to the T-dependent induction of xanthine oxidase seen in animal models [32,33]. Without a doubt, there are further general haemostatic consequences to the influence of androgens on uric acid; however, these just remain to be determined. However, as uric acid affects platelet aggregability equally in both sexes, while plasma uricaemia is characteristically associated with the male sex and blood concentrations of T/DHT, we propose that uric acid-dependent inhibition of platelet aggregation may have distinguishable hormonal triggers in both sexes.

A key novel finding of the present study is that not only T, but also DHT, associates in a significant manner with lipid atherosclerosis markers. Our results show a negative association between concentrations of T/DHT and cholesterol subfractions, and this confirms previous outcomes suggesting that increased concentrations of T/DHT lead to decreased concentrations of total cholesterol and LDL cholesterol [6]. Similar trends in the relationships between total cholesterol or LDL cholesterol and T concentrations have been observed in hypogonadal men treated with T/DHT replacement therapy [4]. This further confirms that T/DHT may have a beneficial impact on lipid profile, even in subjects not receiving hormonal replacement therapies.

Zgliczynski et al. [6] did not note any significant relationship between testosteronaemia and HDL-cholesterol level; in addition, Dobs et al. [34] reported a positive association between the concentration of T and the concentration of HDL cholesterol. However, the present study reveals a negative correlation between these two variables, similarly to relationships described elsewhere among strictly selected (non-drinking and non-smoking) subjects of both sexes [35]. According to some researchers, the decreased oestradiol level and enhanced androgenisation in postmenopausal women results in the creation of an atherogenic lipid profile [36].

Our results might be regarded as inconclusive to an extent, since on the one hand, they suggest that both T and DHT appear to have a cardioprotective impact with regard to the atherogenic total cholesterol and LDL levels, but on the other, they also have proatherogenic properties with regard to the observed HDL cholesterol levels. It is not yet possible to resolve this apparent paradox in a way that accounts for their cardioprotective properties. Hence, further studies are needed with larger separated groups of men and women.

Noteworthy, the significance of the associations between T/DHT and homocysteinaemia and cholesterolaemia disappears after the adjustment for sex, it remains significant only throughout the whole group of subjects. The reason of such an apparent ‘inconsistency’ may be very obvious - the associations revealed for a whole group might have simply resulted from a distinct discrimination of some variables between sexes, but not necessarily from the actual intragroup associations between given variables. In the case of actual associations, we expect that they are maintained also within separate sex subgroups. Evidently, it was not the case for either cholesterol or Hcy. Otherwise, significant associations occur between androgens and fasting glycaemia or the levels of uric acid, and they occur both in the group of subjects as a whole and upon adjustment for sex. Such a relationship favours the conclusion that androgens seem to be strong modulators of glycaemia- and uric acid-dependent atherogenesis, while such effects are apparently much weaker in the case of cholesterol or homocysteine. T replacement therapy in hypogonadal men has been shown to reduce fasting glycaemia [37,38]. Although T has not been observed to have any such a beneficial impact on glucose levels in other tested male subpopulations [39,40], some researchers propose that higher T levels are associated with improved insulin sensitivity and thus, lower glucose levels [41]. On the other hand, androgenaemia has been claimed to positively associate with glycaemia in the group of postmenopausal women [42]. These two opposing lines of evidence imply either negative or positive correlations between glucose levels and androgen concentrations. Our present findings, indicating a positive association between fasting glycaemia and testosteronaemia, favour the latter and contradict the former.

Interestingly, only T and DHT were found to be significantly associated with the level of plasma (anti)atherogenic factors and the markers of platelet activation and reactivity. No significant correlations between E_2_ and the above-mentioned factors occurred. Hence, the results obtained in our *in vitro* tests, showing that E_2_ is also a potent anti-aggregatory factor, are not in line with the *ex vivo* findings, in which E_2_ levels are not related in any way with any of the tested markers of atherosclerosis.

This obvious inconsistency is not so surprising after a careful look at the concentrations used in the *in vitro* study and those noted *ex vivo*. The lowest concentration of E_2_ tested *in vitro* corresponds to the levels of E_2_ found in older men and women in our study, and it should be realised that E_2_ levels did not differentiate between the sexes. The two higher concentrations of E_2_ used for the *in vitro* incubations correspond to the levels characteristic more for premenopausal women and they are still much higher than those noted in older men and women. Therefore, the concentrations of E_2_ circulating in older subjects are probably too low to exert a strong antiplatelet effect, seen in the study as a statistically significant relationship between the levels of E_2_ and the markers of platelet activation or reactivity.

The aggregation experiments performed after the *in vitro* exposure of blood samples to hormonal stimulation found that T and DHT have antiplatelet activity, which confirms the negative associations between (dihydro)testosteronaemia and the markers of platelet reactivity presented in the clinical part of this study. Taking together, we propose that androgens act as potent antiplatelet agents under *in vitro* and *in vivo* conditions in older men and women. However, it also needs to be noted that our present outcomes, especially the androgen-platelet reactivity associations concerning the clinical part of the study, should be read with necessary caution, as no correlation necessarily implies a causal relationship.

Our findings are the first analyses of the associations between plasma concentrations of androgens and several markers of platelet activation and reactivity in a geriatric subpopulation of men and women. T and DHT demonstrate quite a broad range of anti-atherogenic action: directly, through the attenuation of platelet reactivity and activation, and indirectly, through their associations with other plasma atherosclerosis risk factors. Interestingly, the pattern of the associations between some risk factors and androgens might also imply that they possess a possible atherogenic mode of action. As T and DHT exert an inhibitory action on platelet activation and reactivity *in vitro* and *in vivo* in both men and women, these androgens should be considered ‘haemostatic hormones’. Currently, opinions suggesting that vascular diseases affect men to a greater extent than women and that men are at a higher risk of mortality due to these diseases, are critically discussed. Some studies suggest that women are less aware of cardiovascular problems, that they are treated pharmacologically less aggressively than men and they often remain underdiagnosed or misdiagnosed. All these problems may contribute to lower rate of appropriate diagnosis of cardiovascular diseases in women. Hence, men seem to overrepresent the population at risk, which may further lead to not fully justified conclusion that male sex is more prone to atherosclerosis. It is true that incidences of cardiovascular diseases are more numerous among younger men than among the age-matched premenopausal women. However, we also need to notice that after a critical period of menopause the cardiovascular risk in women increases. Indeed, men exhibit higher prevalence of cardiovascular diseases and develop cardiovascular disease earlier than women, but the incidences of cardiovascular events are reported to occur more often in women [43–47]. Comparison of diabetic men and women show higher relative risk of cardiovascular disease due to worse lipid profile and systemic inflammation in diabetic women than in diabetic men [48]. Some confounding variables, like hypertension, may however, further distort the image: when considering hypertension as a leading cardiovascular risk factor, we have to note that hypertensive men have less favourable lipid profile than hypertensive women [49]. Thus, taking into account social, alimentary and/or biochemical factors, it is very difficult to clearly state that the one of sexes is more vulnerable to atherosclerosis than other, mainly because of natural differences in the baseline characteristics of the investigated groups. Our own results presented herein, support the idea that women after menopause appear to be more prone to atherosclerosis than the age-matched men, and the significant contributors include the reduced concentrations of oestrogens and androgens in older women. Since sex hormones are not the only factors that orchestrate the risk of atherogenesis, it seems quite likely that – under some circumstances, their advantageous impact(s) may simply not counterbalance the effects of other proatherogenic risk factors. Certainly, more detailed investigations of these relationships should be undertaken in larger groups of subjects, together with model experiments, to identify the molecular background of the revealed relationships.

## MATERIALS AND METHODS

### Chemicals

LC–MS grade, acetonitrile (MeCN), methanol (MeOH), tetrahydrofuran (THF), dimethyl sulfoxide, MS grade formic acid (HCOOH) and all the analytical standards of testosterone, dihydrotestosterone, α-estradiol, β-estradiol, methyltestosterone, as well as the hormone preparations used for *in vitro* measurements of platelet activation/reactivity were obtained from Sigma (St. Louis, MO, USA) and had a minimum purity specification of 99%. Nitric acid (HNO_3_) was purchased from POCH (Gliwice, Poland). Dichloromethane (DCHM, HPLC grade) was provided by VWR International (Radnor, PA, USA).

PBS was from Avantor Performance Materials Poland S.A. (Gliwice, Poland). Arachidonate, equine tendon collagen and ADP were from Chrono-Log Corp. (Havertown, PA, USA). Fluorolabelled monoclonal antibodies (moAbs): anti-CD61/PerCP, antiCD62/PE, PAC-1/FITC, isotype controls IgG/PE and IgM/FITC, as well as CellFix (1% formaldehyde in PBS) were from Becton Dickinson (San Diego, CA, USA). Ultrapure water was obtained from Milli-Q purification system (Millipore, Bedford, MA, USA). Nitrogen (NM32LA Nitrogen Generator, Peak Scientific Instruments, Billerica, MA, USA) was used as a drying gas.

### Ethics statement

All steps of the experiments with the participation of human subjects were undertaken in accordance with the guidelines of the 1975 Helsinki Declaration for human research. The study was approved by the Committee on the Ethics of Research in Human Experimentation at the Medical University of Lodz. A written abstract of the experiment, including detailed information regarding the study objectives, study design, risks and benefits, was given to each of the volunteers in the course of recruitment. Informed written consent was obtained from each individual at the beginning of each experiment.

### Design of the study

This study presents the results obtained in subgroups of volunteers participating in the project entitled “The occurrence of oxidative stress and selected risk factors for cardiovascular risk and functional efficiency of older people in the context of workload”, funded by the Central Institute For Labour Protection – National Research Institute (Warsaw, Poland) and supervised by the Clinic of Geriatrics at Medical University in Lodz (Poland). Participants were recruited through announcements given on local TV, radio and newspapers. Two basic inclusion criteria were: age within the range of 60 to 65 years and the willingness to participate. The recruitment criteria are described in more detail in an earlier report [50]. To ensure approximately equal participation of men and women, a stratified probability sampling algorithm was used. The research group included roughly 320 subjects (approx. equal sex proportions of 160 men and 160 women), aged from 60 to 65 years, who responded to the announcements (source population). For the purpose of the present study, only those who did not use antiplatelet drugs (acetylsalicylic acid, thienopyridines) within three weeks prior to blood withdrawal were selected (target population). Based on a preliminary pilot study, minimal correlation between the cumulative measure of platelet aggregation and plasma androgen concentration was assumed to be at least 0.30-0.32, the statistical test power, i.e. the likelihood of rejecting false null hypothesis, was assumed to be 90%, and the significance level was assumed to be at least 1% [51]. Of the enlisted target population, 82 women and 73 men were randomly selected (simple unrestricted randomization) to create the subgroup for studying sex steroid level.

Our study consisted of essentially two separate approaches: (1) the monitoring of the *in vivo* associations between platelet function and plasma solutes, including androgens and oestrogens, and (2) the monitoring of the *in vitro* effects of selected sex hormones on the agonist-mediated whole blood platelet aggregation. Blood samples from eight to ten randomly-selected individuals participating in the *in vivo* part of the study (approach 1) were used to perform the incubations with the increasing hormone concentrations under *in vitro* conditions (approach 2).

### Blood sampling, isolation of blood plasma, measurements of blood morphology and plasma biochemistry

Blood was taken after overnight fasting and 15 minute rest directly before blood donation. Blood was collected by aspiration either to vacuum tubes (Sarstedt, Nümbrecht, Germany) supplemented with 0.105 mol/l buffered sodium citrate (citrate:blood ratio = 1:9, v/v) for the measurement of platelet activation and reactivity, or to tubes coated with EDTAK_3_ in the case of samples taken for blood morphology analysis, or to tubes without any anticoagulant when the serum needed to be isolated for further biochemical determinations. In all cases, blood was collected from a peripheral vein cannulated with an 18-gauge needle.

Blood morphology parameters were measured with a 5-Diff Sysmex XS-100i haematological analyser (Sysmex, Kobe, Japan) and serum biochemical parameters were evaluated with a DIRUI CS 400 analyser (Dirui, Changchun, China). Homocysteine concentration was measured with an Immulite 2000 XPi analyser (Siemens, Erlargen, Germany).

In order to obtain blood serum, whole blood taken to the tubes without an anticoagulant was incubated for 30 minutes at 37°C and centrifuged (2000 x g/15 min/4°C). Supernatant (serum) was aspirated and used in further analyses.

Blood plasma was obtained from citrated blood (0.1 mol/l citrate, blood:citrate = 9:1 (v/v)), centrifuged immediately after withdrawal (1000 x g/15 min/4°C), and 1 ml aliquots of plasma were immediately frozen at -80°C and used within the following six months.

### Measurements of whole blood platelet aggregation

Platelet aggregability was measured with a Multiplate Analyser impedance aggregometer (Dynabyte, Munich, Germany), in citrated blood following a 10-minute blood reposition at 37°C to avoid the interference of artefactual platelet activation caused by aspiration. “Tranquilized” 300 µl aliquots of whole blood sample were mixed with an equal volume of PBS for three minutes at 37°C. The mixed sample then was supplemented with one of the used agonists: 0.5 mmol/l arachidonate, 1 µg/ml collagen or 10 µmol/l ADP were added to trigger platelet aggregation. The recording of platelet clumping started immediately thereafter and was tracked for at least 15 minutes. The area under the aggregation curve (AUC) and maximal aggregation (A_max_) were used to describe blood platelet aggregability.

### Measurements of the surface membrane markers of platelet activation and reactivity with the use of flow cytometry

Flow cytometric measurements of the activation of circulating platelets (not agonized *in vitro* either arachidonate nor collagen) included the determinations of the expressions of two surface membrane complexes: the activated glycoprotein complex GPIIb/IIIa (receptor for fibrinogen) and P-selectin. Flow cytometric analyses were performed according to the protocol described in greater detail elsewhere [52].

### Preparation of standard solutions and extraction mixture for hormone determinations with the use of the LC-ESI/MS method

Standard solutions of all the analyzed steroids at the concentrations of 10 µg/ml were prepared by diluting stock solutions containing 1 mg of steroid per 1 ml of methanol. The stock standard solutions of steroids were stored at −20°C until analysis, but no longer than one month. The extraction mixture was prepared by mixing 3 ml of THF with 1.2 ml of DCHM, to ensure the THF/ DCHM volumetric ratio of 5:2.

### Conditions of LC-MS analysis with the use of the LC-ESI/MS method

LC–MS analyses were performed on an Agilent 1260 Infinity HPLC system (Agilent Technologies, Santa Clara, CA, USA) that included a binary pump, an autosampler and a UV variable wavelength detector coupled to an Agilent 6120 mass spectrometry MS (Single Quad, Agilent Technologies, Santa Clara, CA, USA). The mass spectrometer was equipped with the electrospray (ESI) ion source and single quadrupole analyzer, operating in a positive ion mode.

Chromatographic separation of the analytes was carried out with the use of a Poroshell 120 EC-C18 column (Agilent Technologies, St. Clara, CA, USA) (3.0 x 100 mm, particle size 2.7 μm), at temperature of 25°C. The chromatographic results were collected using ChemStation software (Agilent Technologies, Santa Clara, CA, USA).

The mobile phase was composed of 0.1% formic acid in a Millipore grade water (eluent A) and 0.1% formic acid in an LC–MS grade acetonitrile (eluent B). The gradient started linearly from 33.1 to 55.1% B for 12 minutes, followed by an increase to 100% B for two minutes, remained at a constant level of 100% B for two minutes, before decreasing to 33.1% for 6 s. Then, the column was re-equilibrated for 3.9 minutes at initial chromatographic conditions. The total analysis run time was 25 minutes. The injection volume was 5 µl and the flow rate of the mobile phase was set at 0.5 ml/minute. The column was thermostated at 25°C, whereas the temperature at the autosampler storage box was 4°C.

The optimized MS parameters were as follows: ion source temperature 350 °C, drying gas (N_2_) flow rate of gas 12 l/min, nebulizer gas (N2) pressure 60 psi and capillary voltage 6 kV. The fragmentor voltage was set individually for each analyzed compound within the range of 100 to 175 V. The selected ion monitoring (SIM) values chosen for quantitative assay development were (m/z): 363 for cortisol, 289 for testosterone, 255 for β-estradiol, 291 for dihydrotestosterone, 361 for aldosterone, 373 and 303, for two internal standards, betamethasone and methyltestosterone, respectively.

### Sample preparation with the use of dispersive liquid-liquid microextraction (DLLME) method for LC-ESI/MS hormone determinations in blood plasma samples

Human blood plasma samples were stored at -80°C until analysis. Before the analysis, samples were thawed at room temperature and deproteinized by mixing equal volumes (200 µl) of human plasma and 0.1 M HNO3, then filled with water to 1 ml, vortexed and centrifuged at 10,000 rpm for seven minutes. The 980 µl aliquots of supernatants were transferred into 1.5 ml Eppendorf tubes and spiked with internal standard solutions in methanol, 10 µl of methyltestosterone and 10 µl of betamethasone, both at concentration of 10 µg/ml was added.

To extract the steroids, 700 µl of the extraction mixture (containing 500 µl of the extraction solvent (THF) and 200 µl of the disperser solvent (DCHM) was rapidly injected into 1 ml of plasma sample. The mixture was shaken vigorously for about 10 s and then frozen for 10 minutes. Next, the emulsion was centrifuged for 10 minutes at 8000 rpm to enable phase separation. A droplet of DCHM (ca. 150 ± 10 µl in volume), containing the analyzed steroids, was collected and evaporated to dryness at 45^o^C using the CentriVap vacuum rotary evaporator (Labconco, Kansas City, Missouri, USA). The residue was dissolved in 100 µl of methanol, moved to a vial insert and submitted for LC-MS analysis.

### Statistical analysis

The data was expressed as mean + SD or median and IQR (interquartile range: lower [25%] to upper quartile [75%]), depending on data distribution (the Shapiro-Wilk test). Heteroscedasticity was verified with the Brown-Forsythe test. As the data distribution and residual distribution were not normal in the case of numerous variables, the data was subjected to Box-Cox transformation for further analyses. The analysis of outliers was performed with the use Grubb’s and Tukey’s tests; the *k* nearest neighbours analysis was employed as the imputation method when the fraction of missing data did not exceed 3% per a given variable. The principal components analysis and quantile-quantile plot were used to visualize possible batch effects. The block ANOVA was used to correct for possible batch effects in single variables. The Student’s t-test for independent samples, Welch test or Mann-Whitney U-test were used as the inference tests, according to assumptions of data normality and homoscedasticity.

The comparisons with the adjustments for dummy or confounding variables were performed using an analysis of covariance (ANCOVA). The nonparametric Spearman partial correlations method was used to adjust for the effect of gender in simple rank associations. Simple nonparametric correlation estimates were validated using the leave-one out (LOO, jackknife or d-jackknife) techniques.

Multiple logistic regression models were used to reason on the outcome prediction, and the goodness of fit for the models was evaluated using the Hosmer-Lemeshow test [53]. Multivariate regression models were used with continuous dependent variables as the outcomes. The multivariate regression models were validated using bootstrap-boosted procedures (1000-10000 iterations). Multiple group comparisons were performed with various models of the analysis of variance, followed by multiple comparisons *post hoc* tests (Tukey’s honest significant difference test or Dunnett’s test, depending on the tested contrast). For multivariate correlations both the partial correlation coefficient (which reflects the association between a given independent variable and a dependent variable, having eliminated the contribution of other associations of this independent variable with other independent variables in the model) and semipartial correlations (which, in turns, reflects the individual contribution of a given independent variable associated with the overall variability of a dependent variable) were evaluated.

Some bivariate and multivariate analyses, performed separately for men and for women, used a resampling approach with replacement adjusted to sample size. This was intended to overcome any bias related to sample size and to standardize the sample sizes of separate sexes to the overall sample size of the studied group.

The variables describing platelet aggregability in response to agonists and platelet surface membrane glycoprotein expressions were subjected to normalization according to the van der Waerden method. Cumulative normal scores through the used agonists were determined for the markers of whole blood aggregometry (A_max_, AUC, A_max_*AUC/1000) and two flow cytometry markers (P-selectin and the activated GPIIb/IIIa complex) (see the description in the ‘*Results*’ section for details); they were then dichotomised according to the median in such a way that the values below or equal to the median (≤Me, rank 0) corresponded to the “lower overall blood platelet reactivity”, while the values above the median (>Me, rank 1) corresponded to the “higher overall blood platelet reactivity”. Such categorical measures of platelet reactivity were accepted as the grouping variables or dichotomous dependent variables in some multivariate analyses (logistic regression, discriminant analysis). The following software was used for statistical analyses: *Statistica* v. 13.1 (Statsoft), *StatsDirect* v.3.0.182, *Resampling Stats Add-in for Excel* v.4, *GraphPad Prism* v.5.
